# Obligatory intracellular bacterium *Anaplasma phagocytophilum* AnkA regulates actin dynamics and spatiotemporal bacterial release

**DOI:** 10.1371/journal.ppat.1014350

**Published:** 2026-06-24

**Authors:** Mingqun Lin, Nan Duan, Yasuko Rikihisa

**Affiliations:** Department of Veterinary Biosciences, College of Veterinary Medicine, The Ohio State University, Columbus, Ohio, United States of America; University of Nebraska Medical Center, UNITED STATES OF AMERICA

## Abstract

*Anaplasma phagocytophilum* is an obligatory intracellular bacterium that causes an emerging infectious disease, human granulocytic anaplasmosis. It undergoes a biphasic developmental cycle inside membrane-bound vacuoles within the host human neutrophils, maturing from a proliferating reticulate cell form to an infectious dense core (DC) form that is subsequently spontaneously released from host cells to initiate a new infection cycle. However, how *A. phagocytophilum* coordinates growth and release is unknown. Here, we found localized cortical F-actin disruption occurs where *Anaplasma-*containing vacuoles abut on the plasma membrane to release bacteria. Disruption of actin filaments by cytochalasin D and latrunculin B induced unrestrained release of almost all intracellular *A. phagocytophilum* from host cells, which were significantly less infectious than spontaneously released bacteria. *A. phagocytophilum* AnkA, a type IV secretion system (T4SS) effector, was found to localize in the cell periphery with cortical F-actin. By immunoprecipitation followed by mass spectrometry, AnkA was found to interact with actin, α-actinin 4 (Actn4) involved in actin cross-linking, and gelsolin for actin filament remodeling. shRNA-knockdown of Actn4 or gelsolin, enhanced release of premature *A. phagocytophilum*. Glutathione S-transferase (GST)-tagged C-terminus of AnkA (AnkA-C) interacted with actin and gelsolin, whereas the N-terminus (AnkA-N) interacted with Actn4. *In vitro* pyrene-actin polymerization assay showed that GST-AnkA-C has stronger actin polymerizing activity than GST-AnkA or GST-AnkA-N. Ectopically expressed GFP-AnkA-N localized to the plasma membrane and induced membrane ruffling, whereas GFP-AnkA-C colocalized with and enhanced stress fiber formation. These results demonstrate that AnkA is the first example of bacterial molecules interacting with gelsolin and Actn4. The result suggests that by colocalizing with cortical F-actin and controlling F-actin dynamics and cross-linking, AnkA regulates spatiotemporal release of *A. phagocytophilum*. The current study unravels a new paradigm of retention/release mechanism of intracellular pathogen regulated by a T4SS effector.

## Author summary

While cortical cytoskeleton serves as a barrier for exocytosis, how exocytic release of intra-vacuolar bacteria occurs through this barrier is poorly understood. Using obligatory intravacuolar bacteria *Anaplasma phagocytophilum* as a model, this study revealed it actively regulates exocytic bacterial release by deploying a type IV secretion effector, AnkA. Abundant AnkA is progressively secreted during bacterial growth and colocalized with cortical actin filaments. AnkA was found to interact with actin and actin regulatory proteins α-actinin 4 and gelsolin, thereby coordinated bacterial growth, cortical actin remodeling, and bacterial exocytosis. Sustained retention of *A. phagocytophilum* in nutritious intracellular niche is essential for bacterial replication, and timely extracellular release prior to demise of infected host cells is required for bacterial spreading for survival. This study revealed remarkable adaptation in obligatory intravacuolar pathogen by evolving a unique AnkA protein, providing a new paradigm of microbial pathogenesis by manipulation of spatiotemporal actin dynamics by a bacterial molecule.

## Introduction

*Anaplasma phagocytophilum* is a small (0.4 ~ 1.5 µm in diameter), pleomorphic gram-negative bacterium that belongs to the family *Anaplasmataceae* in the order Rickettsiales [1,2]. It causes an emerging infectious disease, human granulocytic anaplasmosis (HGA) [3–7]. HGA can manifest as a severe influenza-like illness symptoms including fever, headache, myalgia, anorexia, and chills, and frequently accompanied by leukopenia, thrombocytopenia, anemia, and elevated levels of serum hepatic aminotransferases [5,8]. Currently, no vaccines exist for HGA, and the use of tick repellent is the only way to prevent infection. The broad-spectrum antibiotic doxycycline is the only drug effective for treating HGA, but delayed initiation of therapy, presence of underlying illness, and immunosuppression often lead to severe complications or death [8].

As a fastidious obligate intracellular bacterium, *A. phagocytophilum* is unable to synthesize most amino acids and lacks pathways for glucose utilization and biosynthesis of many metabolic intermediates [9]. Therefore, it must internalize into and proliferate within membrane-bound vacuoles of host cells for survival, which resemble early autophagosomes and exclude endosomal/lysosomal markers [10–14]. Although *A. phagocytophilum* can infect human endothelial cells, its primary site of infection and the hallmark of HGA lies on the remarkable ability to colonize human granulocytes, the primary mediators of inflammation and innate immunity [12,15–17].

Once internalized into human cells, *A. phagocytophilum* undergoes a biphasic developmental cycle that transitions between an infectious small dense-cored cell (DC) form and a non-infectious, large replicative reticulate cell (RC) form [12,18–21]. DC forms can enter and infect neutrophils or endothelial cells, which transition into RC forms and initiate replication [12,22]. Following intracellular proliferation, RC forms develop and mature into DCs within 2–3 days, which are then gradually and spontaneously released from host cells in a lytic or nonlytic fashion, potentially involves multivesicular bodies biogenesis and exosome release [18,23], to initiate a new cycle of infection [12]. Neutrophils typically undergo apoptosis 6–12 hours after their release from the bone marrow [24,25]; although *A. phagocytophilum* infection can delay neutrophil apoptosis for up to 2–3 days [26,27], the timely entry and release or cell-to-cell spreading from short-lived neutrophils are key to *A. phagocytophilum* survival and bacterial pathogenesis for causing systemic disease. However, how obligatory intra-vacuolar bacteria coordinate intracellular replication and extracellular release to survive and spread is poorly understood.

Type IV secretion system (T4SS) is a versatile bacterial secretion system that delivers proteins and protein-DNA complexes into eukaryotic cells [28–30]. T4SS apparatus was identified in the *A. phagocytophilum* genome, and all the components were expressed by the bacterium in infected host cells especially in the RC stage [9,19,31]. To date, six T4SS effector proteins of *A. phagocytophilum* have been identified [32], including three proteins experimentally characterized by our lab: AnkA [33], *Anaplasma* translocated substrate 1 (Ats-1) [34,35], and ER-Golgi exit site protein of *Anaplasma* (EgeA) [36], as well as *Anaplasma* tick effector A (AteA) [37], AFAP (an actin filament-associated *Anaplasma phagocytophilum* protein) [38,39], HGE-14 protein (APH_0455) [40]. Our previous study showed that AnkA, an ankyrin-repeats (Ank)-containing protein of *A. phagocytophilum*, is translocated into the host cell cytoplasm in a T4SS-dependent manner [33]. By binding to Abl-interactor 1 (Abi-1), AnkA was tyrosine-phosphorylated by a non-receptor protein tyrosine kinase Abl-1, and both AnkA and Abl-1 are critical for *A. phagocytophilum* infection [33]. Several reports have also demonstrated that AnkA was involved in recruiting host tyrosine phosphatase SHP-1 through its SH2 domains [41], or regulation of host gene expression in the nucleus [42–46]. However, most AnkA is found in the cell periphery but not in the nucleus [33,41], and both AnkA-interacting proteins Abi-1/Abl-1 are known to regulate dynamics of actin filaments (F-actin) [47–50]. Therefore, in this study, we investigated roles of AnkA and F-actin dynamics in *A. phagocytophilum* infection, with particular focus on intracellular bacterial retention and release.

## Results

### Loss of cortical F-actin at *A. phagocytophilum* release site

**Fig 1:**
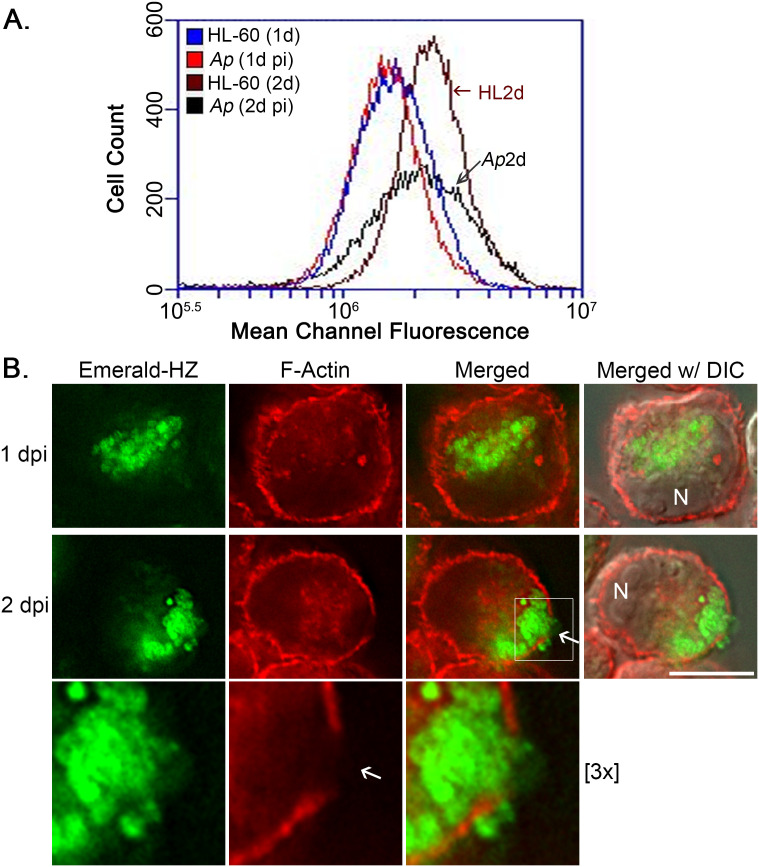
*A. phagocytophilum* infection reduced cortical actin amount. (**A**) Flow cytometry analysis of F-actin contents. *A. phagocytophilum* (*Ap*)-infected or uninfected HL-60 cells at 1 d and 2 d pi were labeled with AF647-conjugated phalloidin and analyzed by a BD Accuri C6 flow cytometer. Data were representative of three independent experiments with similar results, and statistical analysis was performed using one-way ANOVA. Mean ± standard deviation (SD) of mean fluorescence intensities (MFI, × 10^6^) for each group: HL1d, 1.64 ± 0.05; *Ap*1d, 1.50 ± 0.04, *P* = 0.0744 (HL-60 vs. *Ap* at 1 d pi); HL2d, 2.38 ± 0.08; *Ap*2d, 2.03 ± 0.08, *P* = 0.0003 (HL-60 vs. *Ap* at 2 d pi). (**B**) Spatiotemporal cortical F-actin depolymerization at the site of spontaneous *A. phagocytophilum* release. Emerald green-expressing *A. phagocytophilum* HZ (Emerald-HZ)-infected HL-60 cells at 1 or 2 dpi were fixed, incubated with AF647-conjugated phalloidin to label F-actin (pseudo-colored red), and observed under DeltaVision Microscope. Bottom panels: 3× enlarged boxed area at 2 dpi. Arrows, focal F-actin depolymerization; N, nucleus; DIC, Differential interference contrast. Images were representative of three independent experiments with similar results. Bar, 10 µm.

To determine whether *A. phagocytophilum* infection regulates host actin dynamics, flow cytometry was performed. Using Alexa Fluor 647 (AF647)-labelled phalloidin that specifically binds to F-actin but does not inhibit its functionality [51], we examined fluorescence intensities of F-actin during the infection of *A. phagocytophilum* in human promyelocytic leukocytes HL-60. Results showed that F-actin amount in *A. phagocytophilum*-infected HL-60 cells was significantly reduced at 2-day post infection (dpi) by 15% compared to uninfected cells, but not at 1 d pi (Fig 1A), suggesting *A. phagocytophilum* infection caused F-actin depolymerization at late infection stage. We further used fluorescence microscopy to examine cellular F-actin localizations during *A. phagocytophilum* infection. The results showed that at 1 dpi (exponential growth stage), *A. phagocytophilum*-containing vacuoles were localized near the cell center away from cortical F-actin rings, which form a dense filamentous network that lies immediately beneath the plasma membrane (Fig 1B). However, at 2 dpi (late exponential to stationary growth stage), *A. phagocytophilum*-containing vacuoles were localized at the cell periphery, and some underwent exocytosis to spontaneously release *A. phagocytophilum* (Fig 1B, arrows). Three-dimensional reconstruction of fluorescence images along the *Z*-stacks showed that, in these cells, cortical F-actin disappeared at the site where *A. phagocytophilum* was in the process of spontaneous release (Fig 1B and S1 Movie), suggesting localized F-actin depolymerization could potentially facilitate the release of *A. phagocytophilum* from infected host cells. The results corroborate with the previous report that the cortical F-actin acts as a physical barrier to prevent exocytic vesicles from docking at the plasma membrane, and its disassembly promotes exocytosis in neutrophils [52,53].

### Inhibition of actin polymerization induced uncontrolled *A. phagocytophilum* release

**Fig 2:**
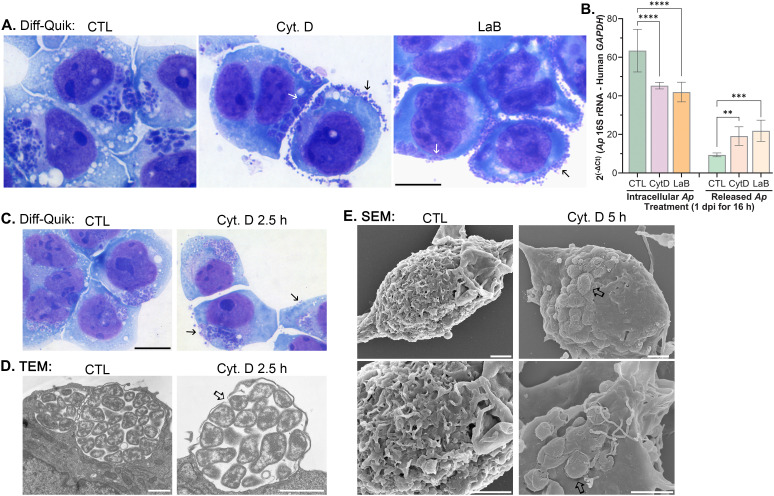
Inhibition of actin polymerization induced massive *A. phagocytophilum* release. **(A, B)** *A. phagocytophilum*-infected HL-60 cells at 1 dpi (~70% infectivity) were treated with 10 µM cytochalasin D (Cyt. **D)**, 1 µM latrunculin B (LaB), or DMSO solvent control (CTL) for 16 **h. (A)** Diff-Quik staining. White arrows, *Anaplasma*-containing vacuoles in the process of exocytosis; Black arrows, released bacteria remaining associated with infected host cells. Images were representative of at least three independent experiments with similar results. Bar, 10 µm. **(B)** Samples following treatment were subjected to differential centrifugation to harvest *A. phagocytophilum*-infected HL-60 cells (Intracellular *Ap*) and the released bacteria from the culture supernatant (Released *Ap*). DNA was extracted and analyzed by qPCR using primers for *A. phagocytophilum* 16S rRNA and normalized against human GAPDH gene. Data were presented as the mean ± standard deviation (SD) from two independent experiments. ** *P*  <  0.01; *** *P*  <  0.001; **** *P*  <  0.0001; one-way ANOVA. (**C, D, E**) *A. phagocytophilum*-infected HL-60 cells at 2 dpi (~90% infectivity) were treated with 10 µM cytochalasin D for 2.5 h (**C, D**) or 5 h **(E)**. Aliquot of samples were subjected to **(C)** Diff-Quik staining (bar, 10 µm) or processed for observation under transmission or scanning EM (**D**, TEM; **E**, SEM; bar, 2 µm). Arrow in **(D)**, protrusion of vacuoles with disrupted plasma membrane; Arrows in **(E)**, protrusions corresponding to the size of *A. phagocytophilum* (0.5–1 µm). **(E)** SEM magnifications: upper panels, 8,000×; lower panels, 16,000×.

To analyze the roles of F-actin dynamics on *A. phagocytophilum* intracellular retention and release, we used chemical inhibitors that disrupt host cell actin polymerization. Cytochalasin D binds to the barbed ends of actin monomers, therefore, inhibits actin polymerization and promotes F-actin depolymerization [54]; whereas Latrunculin B sequesters actin monomers, thus, inhibits F-actin polymerization and destabilizes the entire actin cytoskeleton [55]. In the control groups, *A. phagocytophilum*-containing vacuoles were evenly distributed in the cytosols in infected HL-60 cells. However, treatment of *A. phagocytophilum*-infected HL-60 cells with cytochalasin D or latrunculin B for 16 h at 1 dpi induced exocytosis of intracellular *A. phagocytophilum* (Fig 2A): a massive amount of bacteria were either present at extracellular spaces or remained attached to the cell surface, while *Anaplasma*-containing vacuoles were located near or docked at the cell edge, presumably in the process of exocytosis (Fig 2A).

Quantitative PCR (qPCR) analysis showed that F-actin disruption induced the release of significant amount of *A. phagocytophilum* from infected HL-60 cells into the culture supernatant; consequently, intracellular bacteria numbers were significantly reduced following F-actin disruption in the infected cells (Fig 2B). The enhanced extracellular release of *A. phagocytophilum* was not due to cell lysis or cell death, as no significant differences in the viabilities of host cells and *A. phagocytophilum* were observed among DMSO solvent control, cytochalasin D, and latrunculin B-treated groups (S1 Fig). This was confirmed using a BacLight Live/Dead Viability Kit, which monitors the viability of bacteria and cell populations based on the membrane integrity of the cells (S1A–S1B Fig), or the CyQUANT MTT (3-[4,5-dimethylthiazol-2-yl]-2,5 diphenyl tetrazolium bromide) cell viability assay, which measures the conversion of MTT into formazan crystals by redox potential in viable mammalian cells (S1C Fig).

Time-course studies with latrunculin B treatment of *A. phagocytophilum*-infected HL-60 cells showed that as early as 2 h post-treatment (hpt), *Anaplasma*-containing vacuoles were detected at the cell edge (S2A Fig, white arrows), and individual bacteria were present at the extracellular spaces (S2A Fig, black arrows). Significant numbers of *A. phagocytophilum* (more than 30 per cell) were detected at extracellular spaces and remained bound to the cell surface of infected HL-60 cells from 16–22 hpt (S2B Fig); and at 24 hpt, most *A. phagocytophilum* were released from the cells with approximately 10–20 bacteria remaining attached on the cell surface (S2B Fig), resulting significantly reduced numbers of intracellular bacteria (S2B Fig). For the control group, most *Anaplasma*-containing vacuoles remained intracellular at 1–2 dpi with less than five released *A. phagocytophilum* at the extracellular spaces (S2A–S2B Fig). Although microtubules regulate neutrophil exocytosis [56,57], nocodazole, which promotes depolymerization of microtubules [58], had no significant effects on bacterial infection or release (S3 Fig).

Light and transmission electron microscopy showed that at 2 dpi, some *Anaplasma*-containing vacuoles were docked on the plasma membrane, and cytochalasin D treatment for 2.5 h caused protrusion of vacuoles with disrupted plasma membrane to release bacteria (Fig 2C–2D, and S2C Fig). Scanning electron microscopy (SEM) of CytD-treated cells is known to reveal profound surface remodeling due to actin depolarization, characterized by significant loss or reduction of membrane protrusions, resulting in a smooth or featureless plasma membrane [59]. Our SEM images demonstrated that the plasma membrane of *A. phagocytophilum*-infected HL-60 cells at 2 dpi exhibited extensive ruffling structures with irregular protrusions and lamellipodia-like folds [60] (Fig 2E). In contrast, cytochalasin D treatment led to the disappearance of membrane ruffles and lamellipodia (Fig 2E). Notably, SEM images showed unusual clusters of cell surface protrusion corresponding to the size of *A. phagocytophilum* (0.5-1 µm) following CytD-treatment of infected cells (Fig 2E, arrows), supporting our data of light microscopy and TEM.

### *A. phagocytophilum* released by F-actin depolymerization is poorly infectious

**Fig 3:**
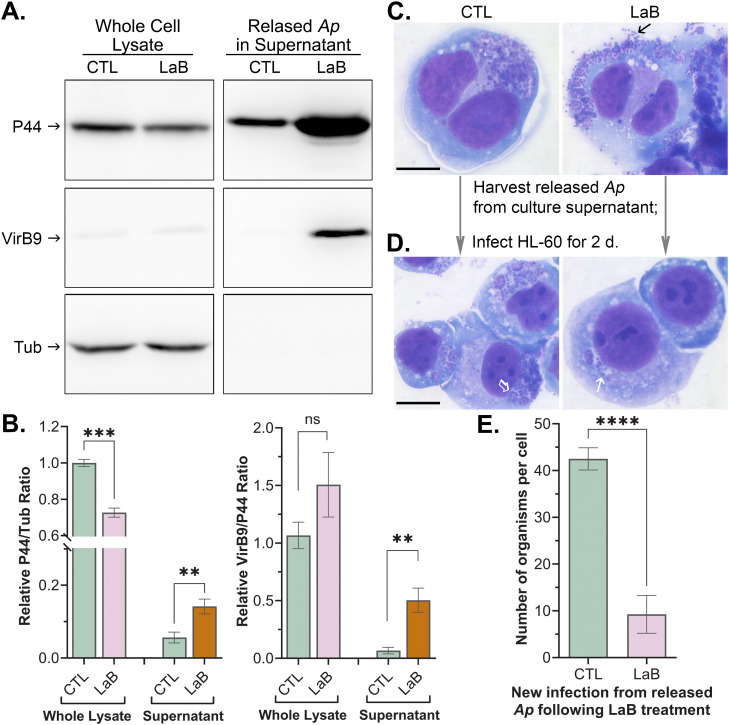
*A. phagocytophilum* released by F-actin depolymerization is poorly infectious. (**A–C**) *A. phagocytophilum* (*Ap*)-infected HL-60 cells were treated with 1 µM latrunculin B (LaB) or DMSO solvent control (CTL) at 1 dpi for 16 h. Samples were centrifuged and released *Ap* were harvested from culture supernatants. (**A**) Pellets from 2 × 10^6^ cells were lysed in 400 µl of RIPA buffer (designated as whole cell lysates) and released *Ap* in the supernatant were lysed in 40 µl of RIPA buffer. Aliquots of samples (1/20 for whole cell lysates and 1/2 for supernatants) were subject to Western blot analysis using antibodies against *Ap* P44 and VirB9, or human Tubulin (Tub). (**B**) Band densities from three independent experiments were quantitated by ImageQuaNT, and P44 amounts were normalized against Tubulin in whole cell lysates, whereas VirB9 were normalized against P44 in the corresponding samples. The relative ratios were further normalized with the total sample volumes, and those of CTL groups in the whole cell lysates were set as 1. (**C**) Representative of CTL and LaB-treated cells by Diff-Quik staining. Arrow, extracellular released bacteria. Bar, 10 µm. (**D, E)** Released *Ap* were harvested from culture supernatants following treatment with latrunculin B (LaB) or DMSO solvent control (CTL) at 1 dpi for 16 h (**C**) and used to infect naïve HL-60 cells. (**D**) Representative images of new infection at 2 dpi, Diff-Quik staining. Open arrow, multiple large *Ap*-containing vacuoles in infected HL-60 cells; solid arrow, small vacuoles containing 1–2 *Ap*. Bar, 10 µm. (**E**) Quantitation of numbers of *Ap* organisms in 100 cells at 2 dpi from three independent experiments. (**B, E**) Data are presented as the mean ± SD from three independent experiments, and statistical analysis was performed by unpaired Student’s *t* test. ** *P* < 0.01; *** *P* < 0.001; **** *P* < 0.0001; ns, not significant.

Since cytochalasin D and latrunculin B have similar effects on disrupting the actin cytoskeleton and inducing extracellular release of *A. phagocytophilum* (Fig 2A–2B), latrunculin B alone was used to evaluate the infectivities of released bacteria. Western blot analysis using antibodies against *A. phagocytophilum* major outer membrane protein P44 [61,62] showed that, similar to qPCR analysis (Fig 2B) and direct counting of bacterial numbers (S2B Fig), latrunculin B treatment significantly reduced intracellular bacterial amounts, but increased extracellular release of *A. phagocytophilum* in the culture supernatant (Fig 3A–3B). Because *A. phagocytophilum* VirB9 (one of core proteins of Type IV secretion apparatus) is strongly expressed by replicating *A. phagocytophilum* (RC form) and nearly undetectable in spontaneously released mature bacteria (infectious DC form) [19], we examined the amount of VirB9 proteins in released *A. phagocytophilum*. VirB9 proteins were almost undetectable in intracellular and spontaneously released bacteria in control groups at 2 d pi, or in intracellular bacteria following latrunculin B-treatment (Fig 3A–3B). However, strong VirB9 protein bands were detected in released *A. phagocytophilum* following latrunculin B-treatment (Fig 3A–3B), suggesting that these released bacteria by F-actin depolymerization were mainly replicating RC forms, not mature DC forms.

To examine infectivity of the released *A. phagocytophilum*, bacteria were freshly harvested from culture supernatants of infected cells following DMSO control or latrunculin B-treatment and used to infect naïve HL-60 cells for 2 d (Fig 3C–3E). Although the amount of *A. phagocytophilum* released into culture media was significantly greater (approximately 3-fold) in latrunculin B-treated group (Fig 3A–3B), they were significantly less infectious compared to spontaneously released bacteria (Fig 3D–3E). These data suggest that F-actin disruption induced premature release of RC forms, which are less infectious compared to spontaneously released DC forms.

### *A. phagocytophilum* AnkA localizes in cell periphery and interacts with actin, gelsolin, and α-actinin 4

**Fig 4:**
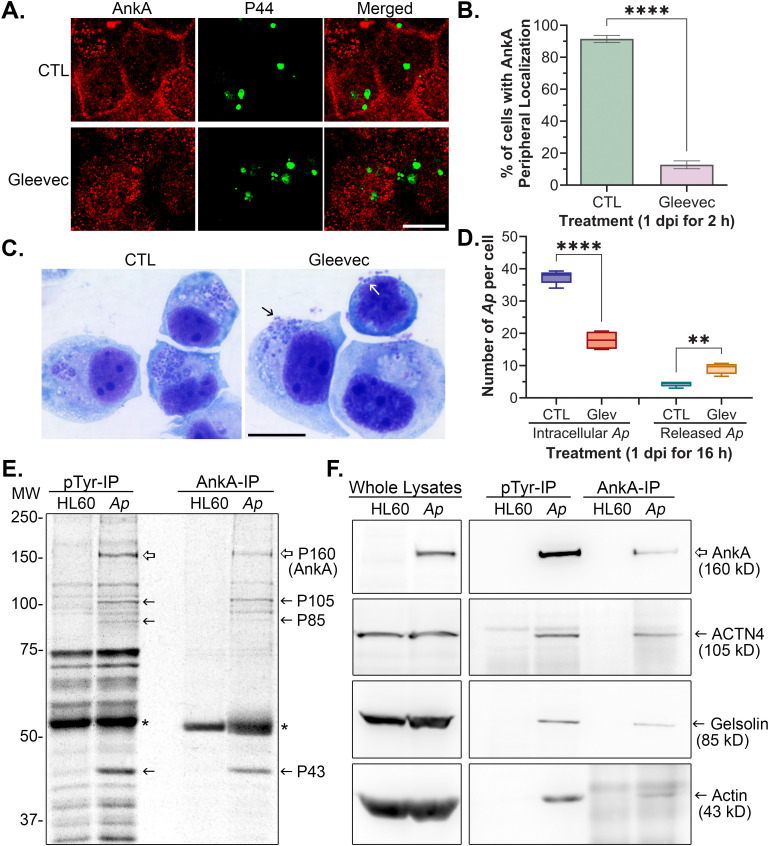
*A. phagocytophilum* AnkA localizes at the cell periphery and interacts with actin, gelsolin, and α-actinin 4. (**A–B**) AnkA localization at the cell periphery requires tyrosine phosphorylation by Abl-1 kinase. *A. phagocytophilum* (*Ap*)-infected HL-60 cells at 1 dpi were treated with DMSO solvent control (CTL) or 20 µM Gleevec for 2 h. Cells were labeled with mouse anti-*Ap* P44 and rabbit anti-*Ap* AnkA IgG, followed by AF488–anti-mouse and AF555–anti-rabbit secondary Abs. (**A**) Representative image of confocal microscopy. Bar, 10 µm. (**B**) Quantitation of numbers of cells with peripheral localization of AnkA in *Ap*-infected cells (>100 cells from three independent experiments). **** *P* < 0.0001, unpaired Student’s *t* test. **(C–D)** Gleevec induces extracellular *Ap* release. *Ap*-infected HL-60 cells at 1 dpi (~70% infectivity) were treated with 20 µM Gleevec (Glev) or solvent control (CTL) for 16 h. Black arrow, released bacteria remaining associated with infected host cells; white arrow, *Ap*-containing vacuoles in the process of release. (**C**) Diff-Quik staining. Bar, 10 µm. (**D**) Numbers of *A. phagocytophilum* inside HL-60 cells (Intracellular *Ap*), or individual bacteria either present at extracellular spaces or remain attached to the cell surface (Released *Ap*) were quantitated by counting approximately 80–100 cells from three independent experiments. ** *P* < 0.01; **** *P* < 0.0001; unpaired Student’s *t* test. (E-F) AnkA interacts with human actin, gelsolin, and Actn4. Uninfected or *A. phagocytophilum* (*Ap*)-infected HL-60 cells at 3 dpi were lysed in RIPA buffer and immunoprecipitated (IP) with mouse anti-phosphotyrosine (pTyr) mAb or rabbit anti-AnkA IgG. (**E**) Samples were subjected to SDS-PAGE and stained with GelCode Blue. Four distinct protein bands at 160 (AnkA, open arrows), 105, 85, and 43 kDa that were only detected in infected cells (indicated by arrows) were excised for protein identification by mass spectrometry. MW, protein molecular weight marker (kDa); * IgG heavy chain. (**F**) Whole cell lysates or immunoprecipitated samples were subjected to Western blot analysis using specific antibodies against proteins identified by proteomics, including AnkA, Actn4, Gelsolin, and Actin.

AnkA, a T4SS effector of *A. phagocytophilum*, directly binds to Abi-1, an adaptor protein of Abl-1 tyrosine kinase, and is tyrosine-phosphorylated by Abl-1 [33]. In *A. phagocytophilum*-infected HL-60 cells, the majority of AnkA proteins were localized in the cell periphery (Fig 4A–4B, control) at 1 d pi where cortical F-actin is localized (Fig 1). Abl kinase inhibitor Gleevec [63–65], which inhibits AnkA tyrosine phosphorylation [33], significantly reduced cortical localization of AnkA (Fig 4A–4B), suggesting that tyrosine phosphorylation of AnkA by Abl-1 kinase is critical for AnkA localization in the cell periphery. Furthermore, Gleevec treatment at 1 dpi for 16 h, caused *A. phagocytophilum*-containing vacuoles to localize near cell periphery and significantly induced bacterial release from host cells (Fig 4C–4D), suggesting that AnkA and tyrosine phosphorylation might be involved in the regulation of actin dynamics and bacterial release. We therefore performed immunoprecipitation using antibodies against AnkA or phospho-tyrosine with lysates of *A. phagocytophilum*-infected HL-60 cells, which pulled-down and enriched the tyrosine-phosphorylated AnkA protein at 160 kDa (P160, Fig 4E) as demonstrated previously [33]. In addition, three protein bands at 105, 85, and 43 kDa were co-immunoprecipitated with the tyrosine-phosphorylated AnkA (Fig 4E). Mass spectrometry identified these proteins as human α-actinin-4 (Actn4, P105), gelsolin (P85), and actin (P43), respectively, which were confirmed by Western blot analysis using respective antibodies (Fig 4F).

### Knockdown of Actn4 and gelsolin enhanced extracellular release of *A. phagocytophilum*

**Fig 5:**
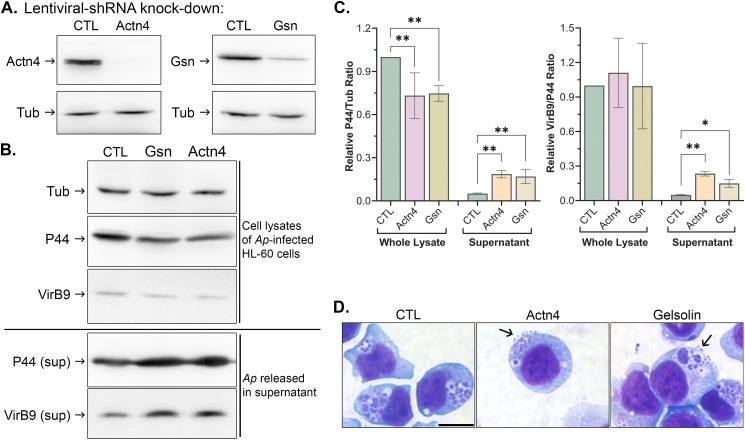
shRNA knockdown of gelsolin and Actn4 inhibits *A. phagocytophilum* infection and release. HL-60 cells were transduced with Lentiviral-shRNA particles targeting human ACTN4, gelsolin (Gsn), or non-human control (CTL). Positively transduced cells were selected with puromycin and infected with host cell-free *A. phagocytophilum* (*Ap*) at MOI of 50. At 2 dpi, infected cells were collected and released *Ap* were harvested from culture supernatants (Sup). (**A–B**) Aliquots of samples (1/20 for whole cell lysate; 1/2 for supernatant) were subjected to Western blotting using antibodies against human Actn4, Gsn, or Tubulin (Tub), and *Ap* P44 or VirB9. (**C**) The band densities were quantitated by ImageQuaNT, and P44 amounts were normalized against Tubulin corresponding to the total sample volumes, whereas VirB9 were normalized against P44 in the corresponding sample. The relative ratios for CTL groups in the whole cell lysate were set as 1. Data are presented as the mean ± SD from three independent experiments. Significantly different from the control group by one-way ANOVA (* *P*  <  0.05; ** *P*  <  0.01). (**D**) Representative *Ap*-infected HL-60 cells transduced with CTL, Actn4, or Gsn shRNA at 2 dpi, Diff-Quik staining. Arrow, released bacteria remaining associated with infected host cells. Bar, 10 μm.

AnkA-interacting proteins Actn4 and gelsolin are actin-regulatory proteins that involved in the modulation of F-actin dynamics at the cell surface or in the cytosol [66,67]. Actn4 is a spectrin superfamily protein involved in binding F-actin to the cell membrane and essential for cell structure, cell motility, and signal transduction [67–69]. Gelsolin is an F-actin severing and capping protein that regulates actin filament remodeling [66,70]. We, therefore, examined the roles of Actn4 and gelsolin on *A. phagocytophilum* release. Using lentiviral vectors expressing shRNAs targeting human Actn4 and gelsolin, transduction of HL-60 cells with these vectors was efficient and generated stable cell lines that significantly reduced Actn4 or gelsolin protein expressions compared to scrambled shRNA controls (Fig 5A). Knockdown of Actn4 or gelsolin in HL-60 cells significantly reduced intracellular *A. phagocytophilum* as demonstrated by lower amount of P44s in infected HL-60 cells at 2 dpi (Fig 5B–5D), and enhanced the release of VirB9-positive premature *A. phagocytophilum* into culture supernatant from infected host cells, with a portion of released bacteria remained associated with infected host cells similar to those treated with F-actin disruption reagents (Fig 5B–5D). These data imply that Actn4 and gelsolin are required to retain *A. phagocytophilum* in HL-60 cells.

### AnkA promotes actin polymerization through its N-terminal interaction with Actn4 and C-terminal interactions with gelsolin and actin

**Fig 6:**
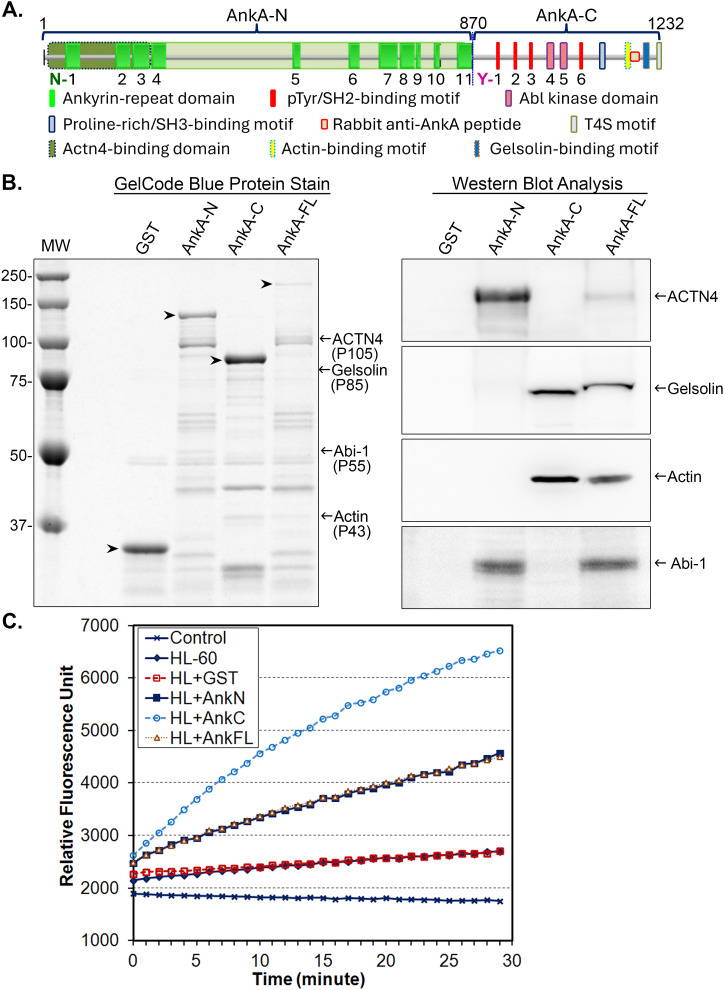
AnkA promotes actin polymerization via AnkA-N interaction with Actn4 and AnkA-C interactions with gelsolin and actin. **(A)** Predicted AnkA domain structure. Protein motifs of AnkA were analyzed by NCBI Conserved Domain (https://www.ncbi.nlm.nih.gov/cdd), UniProt (https://www.uniprot.org), and ScanSite 4 (https://scansite4.mit.edu). AnkA consists of 11 Ank-repeats (green boxes, # N-1–11) clustered at the N-terminus (AnkA-N, 1–870 aa), 6 tyrosine-phosphorylation motifs (pTyr/SH2-binding motifs, red boxes), and 1 proline-rich domain (SH3-binding motif, blue box) near C-terminus (AnkA-C, 871–1,232 aa). Predicted tyrosine-phosphorylation motifs by Src family tyrosine kinases are shown in red boxes (AnkA-C: # Y-1, 2, 3, and 6) and Abl-1 kinase in red boxes with blue outlines (AnkA-C: # Y-4 and Y-5). Yellow square box with red outline, peptide sequences recognized by rabbit anti-AnkA IgG (1,143–1,162 aa); orange box, the C-terminal T4S consensus motif. AlphaFold3 prediction of AnkA domains or motifs interaction with actin-regulatory proteins (S4 Fig): ACTN4, olive-colored box (5–192 aa); β-Actin, yellow (1,133–1,144 aa); Gelsolin, blue (1,169–1,192 aa). **(B)** Recombinant GST-AnkA proteins interact with Actin, gelsolin, and Actn4 by GST-pull down assay. Equal amounts of purified recombinant GST or GST-AnkA proteins were immobilized on glutathione-agarose columns and incubated with equal amounts of HL-60 cell lysates. Bound proteins were eluted by glutathione-elution buffer, and subjected to protein staining by GelCode blue or Western blotting with specific antibodies. Arrowheads, rGST-tagged fusion proteins; Arrows, interacting human proteins by GST-pulldown assay. AnkA-N, -C, and -FL proteins corresponded to N-, C-terminal or full-length AnkA respectively shown in **(A)**. **(C)** Recombinant AnkA proteins enhance actin polymerization kinetics by pyrene-actin assay. Equal molar amounts of recombinant GST or GST-AnkA proteins (1 μM) were mixed with 1 µg of pyrene-actin and HL-60 cell lysate as host actin nucleation factors. Actin polymerization was measured as arbitrary fluorescence intensity of pyrenyl F-actin (Ex_365nm_/ Em_407nm_) in each reaction mixture and plotted versus time following the addition of actin polymerization buffer. Control, pyrene-actin only in polymerization buffer; HL-60, buffer containing only pyrene-actin and HL-60 cell lysate. Data were the representative of two independent experiments with similar results.

Analysis of conserved domains and motifs of AnkA protein indicates that it mainly consists of 11 ankyrin-repeats clustered at the N-terminus (AnkA-N, 1–870 AA) and 6 tyrosine-phosphorylation motifs by Src or Abl-1 family kinases at the C-terminus (AnkA-C, 871–1,232 AA) (Fig 6A). In addition, there are a proline-rich/SH3 binding motif and the consensus secretory motif for T4S apparatus at the C-terminus (Fig 6A). To determine which AnkA domains interact with actin, gelsolin, or Actn4, recombinant glutathione S-transferase (GST)-tagged full-length, N-, or C-terminus of AnkA proteins were constructed. GST pull-down assay showed that AnkA-N interacted with Actn4, whereas AnkA-C interacted with actin and gelsolin (Fig 6B). Abi-1, which was previously identified to bind AnkA directly [33], was found to interact with the N-terminus of AnkA (Fig 6B). Full-length AnkA (AnkA-FL) could bind to all four interacting partners, but the control GST protein did not interact with any of these proteins (Fig 6B).

Since AnkA binds actin and actin regulatory proteins, the actin polymerization activity of AnkA and its N-and C-terminus were tested by an *in vitro* polymerization assay using pyrenyl actin, a fluorescent derivative of actin that exhibits much higher fluorescence intensity when present as F-actin than as G-actin [71,72]. Using HL-60 cell lysate as a source of cellular factors [72], *in vitro* pyrene-actin polymerization assay showed that recombinant GST-AnkA proteins significantly enhanced actin polymerization compared to control GST proteins (Fig 6C). Interestingly, AnkA-C that binds actin and gelsolin (Fig 6B) had higher activity than AnkA-FL or AnkA-N in inducing actin polymerization *in vitro* (Fig 6C).

To gain better understanding of *A. phagocytophilum* AnkA interactions with host proteins, three-dimensional (3D) protein structures of AnkA and protein-protein interactions were analyzed by AlphaFold 3 [73]. 3D structure prediction showed that AnkA-N consists of mostly helix-loop-helix motifs, characteristics of ankyrin-repeats, whereas AnkA-C contains mainly intrinsically disordered regions except for a short α-helix motif (S4A Fig). The first three ankyrin-repeats are predicted to interact with Actn4 (S4B Fig), while the short α-helix motif of AnkA-C is predicted to interact with gelsolin (S4C Fig). Interestingly, AnkA interaction with actin induces the formation of an additional α-helix motif (residues 1,133–1,144, S4D Fig), which precedes the gelsolin-binding α-helix (residues 1,169–1,192).

### AnkA regulates F-actin dynamics and promotes *A. phagocytophilum* infection

**Fig 7:**
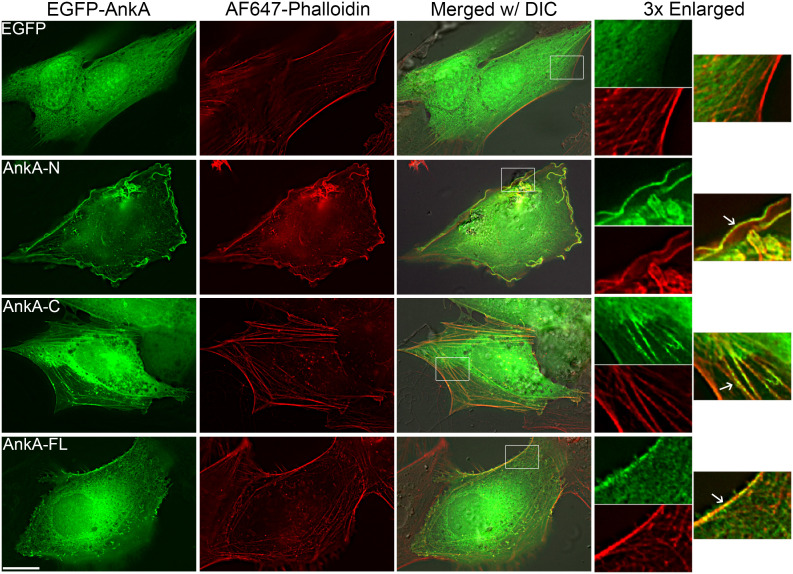
Ectopically expressed EGFP-AnkA and EGFP-AnkA-N localize to cortical F-actin, whereas EGFP-AnkA-C localizes to stress fibers. RF/6A cells were transfected with EGFP control or EGFP-AnkA plasmids using Fugene HD reagent for 1 d, labeled with AF647-Phalloidin (pseudo-colored red), and examined under DeltaVision deconvolution microscope. Right panels, 3× enlargement of box areas in merged images; DIC, Differential interference contrast. Arrows indicate colocalization of EGFP-AnkA with F-actin. Bar, 10 µm.

To determine the cellular distribution of AnkA and its effects on actin dynamics, we used thinly spread, adherent monkey endothelial RF/6A cells, which allows unambiguous localization of cellular molecules [74] and can be infected with *A. phagocytophilum* [15]. We therefore transfected RF/6A cells with EGFP-tagged full-length, N- or C-terminus of AnkA to study AnkA functions. Fluorescent microscopy revealed that EGFP-AnkA colocalized with F-actin as labeled by AF647-phalloidin, and notably, N- or C-terminal AnkA had distinct effects on F-actin distribution (Fig 7). Line profile analysis showed that stronger fluorescence intensities of AnkA-N and AnkA-FL were detected and colocalized with the cortical F-actin at the cell edge; whereas stronger fluorescence intensities of AnkA-C were detected and colocalized with actin stress fibers in the cytosolic regions (Fig 7 and S5 Fig). In addition, extensive membrane ruffling and blebbing were observed in AnkA-N-transfected cells (Fig 7). Since ACTN4 is an F-actin crosslinking protein that binds and anchors actin filaments to the plasma membrane, especially in cell protrusions and focal adhesions [68,75,76], the interaction of AnkA-N with ACTN4 is likely required for AnkA colocalization with cortical F-actin, validating the interaction of N- and C-terminus of AnkA with actin and actin-regulatory proteins (Fig 6 and S4 Fig).

**Fig 8:**
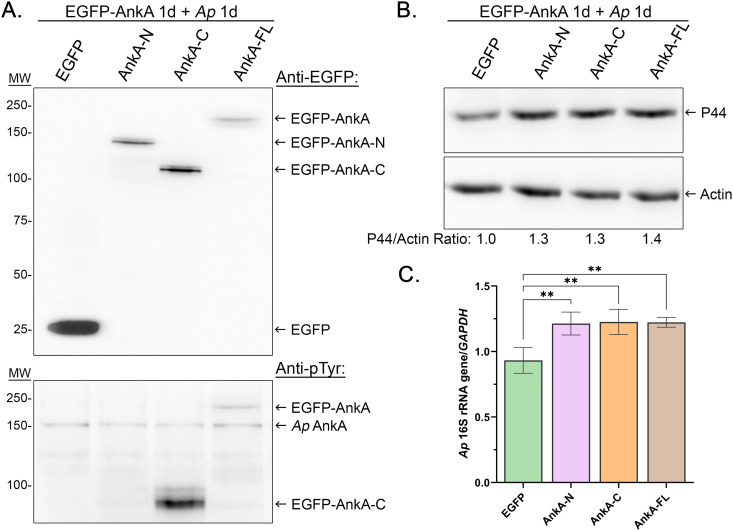
Ectopically expressed EGFP-AnkA proteins enhance *A. phagocytophilum*-infection in RF/6A cells. RF/6A cells were transfected with EGFP-AnkA plasmids by Fugene HD for 1 d, and infected with *A. phagocytophilum* (*Ap*) for 1 **d. (A–B)** Samples were subject to Western blot analysis using antibodies against EGFP and phosphotyrosine (pTyr) **(A)**, or human Actin and *Ap* P44 **(B)**. *Ap* AnkA, native AnkA expressed by *A. phagocytophilum* **(A)**. Band densities were quantitated by ImageQuaNT, and ratios of P44 normalized to Actin were shown under each lane **(B)**. **(C)** Alternatively, RNA samples were extracted from EGFP-AnkA-transfected, *A. phagocytophilum*-infected RF/6A cells at 2 dpt (1 dpi) and analyzed by RT-qPCR using primers for *A. phagocytophilum* 16S rRNA and normalized against RF/6A *GAPDH* gene. Data are presented as the mean ± SD from three independent experiments. ** *P*  <  0.01, significantly different by one-way ANOVA.

Genetic manipulation, especially targeted mutagenesis, remains challenging for obligatory intracellular bacteria due to their genetic intractability, including the difficulties in DNA delivery while retaining bacterial viability, efficient reintroduction of the transformed bacterial population into host cells, limited selection markers, and the limited efficiency of homologous recombination and transposition systems [77,78]. Furthermore, essential genes required for bacterial survival cannot be knocked out, as we previously demonstrated that, by delivering anti-AnkA IgG by Chariot protein delivery system, AnkA is required for *A. phagocytophilum* infection [33]. Therefore, we further analyzed F-actin distribution and dynamics regulated by ectopically expression of EGFP-tagged FL-, N-, or C-terminal AnkA proteins in mCherry-expressing *A. phagocytophilum-*infected cells [79]. The results demonstrated that the cellular distribution of actin dynamics regulated by EGFP-AnkA proteins in *A. phagocytophilum-*infected cells was similar to that in the uninfected cells (S6 Fig). The ectopic expression of EGFP-tagged FL-, N-, or C-terminal AnkA proteins was detected by Western blot analysis using anti-GFP antibody in transfected RF/6A cells infected with *A. phagocytophilum* (Fig 8A). In addition to native AnkA expressed by *A. phagocytophilum*, EGFP-AnkA-FL and EGFP-AnkA-C that contain tyrosine kinase phosphorylation sites were also tyrosine-phosphorylated in RF/6A cells (Fig 8A). Western blotting and quantitation of ratios *A. phagocytophilum* major outer membrane protein P44 vs. human Actin, or quantitative RT-PCR based on *A. phagocytophilum* 16S rRNA, all demonstrated that ectopically expressed AnkA proteins significantly increased intracellular *A. phagocytophilum* (Fig 8B–8C), indicating that modulation of actin dynamics by AnkA facilitates overall bacterial yield.

### Actin dynamics are critical for overall *A. phagocytophilum* infection of host cells

Foregoing results showed actin dynamics and AnkA are critical for spatiotemporal regulation of *A. phagocytophilum* release from infected cells, thus overall yield of infectious bacteria (summarized in Table 1), and a previous study showed that disruption of actin cytoskeleton in cytochalasin D-pretreated HL-60 cells blocks bacterial *A. phagocytophilum* uptake [41]. To further understand roles of cytoskeleton dynamics on *A. phagocytophilum* internalization, proliferation, and release, we tested additional cytoskeleton-disrupting agents. The actin polymerization promoting factors, Rac/WAVE/Rho GTPase and N-WASP (neuronal Wiskott–Aldrich syndrome protein), are known to control the assembly and disassembly of the actin cytoskeleton in responses to extracellular signals [80–82]. Non-muscle myosins like myosin II, the actin-based motors, participate in many cellular functions like endocytosis, exocytosis, and intracellular trafficking [83,84]. Inhibitors that interfere with actin dynamics or actin-based vesicular trafficking, including inhibition of N-WASP activation by Wiskostatin [85], Rho GTPase by C3-transferase [86], myosin light-chain kinase (MLCK) by ML-7 [87], and myosin II ATPase by blebbistatin [88,89], significantly reduced *A. phagocytophilum* entry and proliferation in HL-60 cells, but did not induce bacterial release (Table 1, S7 Fig). However, inhibitors that directly block F-actin formation like cytochalasin D and latrunculin B, and Abl kinase inhibitor Gleevec not only significantly inhibited bacterial internalization and proliferation, but also enhanced bacteria release (Table 1, Figs 2A–2B, 4C–4D, and S7A Fig). In contrast, microtubule dynamics have limited impacts on *A. phagocytophilum* infection. Nocodazole had no effect on *A. phagocytophilum* internalization, proliferation, or release (Table 1, S3 and S7 Fig). Taxol, which promotes assembly of microtubules and inhibits tubulin disassembly thus deforms the cytoskeleton [90], reduced *A. phagocytophilum* infection to a smaller extent as ML-7 (Table 1, S7 Fig).

**Table 1:** Effects of cytoskeleton-disrupting agents on *A. phagocytophilum* entry, proliferation, and release (1).

Inhibitor (abbreviation, concentration)	Targets/Functions	Inhibition on Internalization	Inhibition on Proliferation	Induction of Release	Source
Cytochalasin D (CytD, 10 µM); Latrunculin B (LaB, 1 µM)	Inhibitor of actin microfilament formation	+++	++	+++	EMD Biosciences (San Diego, CA)
C3-Transferase (C3T, 2 µg/ml)	Rho GTPase inhibitor	+++	+++	–	Cytoskeleton, Inc. (Denver, CO)
Blebbistatin (Bleb, 100 µM)	Inhibitor of non-muscle myosin II ATPase activity	++	++	–	EMD Biosciences
Wiskostatin (Wisk, 10 µM)	N-WASP Inhibitor (blocks actin filament assembly)	n/d	++	–	EMD Biosciences
ML-7 (20 µM)	Myosin light chain kinase (MLCK) inhibitor	+	+	–	EMD Biosciences
Gleevec (20 µM)	Abl kinases inhibitor	++	++	+	Norvatis (Basel, Switzerland)
Taxol (10 µM)	Promote assembly of microtubules and inhibits tubulin disassembly	n/d	+	–	EMD Biosciences
Nocodazole (Noc, 5 µM)	Promote tubulin depolymerization	–	–	–	EMD Biosciences

(1) To examine effects of inhibitors on bacterial internalization, naïve HL-60 cells were pretreated with inhibitors for 2 h and infected with host cell-free *A. phagocytophilum* (*Ap*) for 4 h in the presence of inhibitors, then washed to remove unbound or uninternalized bacteria. To determine effects of inhibitors on bacterial infection or proliferation, Ap-infected HL-60 cells at 1 d pi were treated with inhibitors for 16 h. At 2 dpi, cells were stained by Diff-Quik method, and infectivities were quantitated under the microscope. Abbreviation and working concentration of inhibitors used in this study were shown in parenthesis. Effects of inhibitors on bacterial entry, proliferation, or release were summarized based on Figs 2, S2, S3, and S7: +++, > 80%; ++, > 50%; + , > 20%; -, no effects; n/d: not determined.

## Discussion

As an obligatory intracellular bacterium of short-lived human neutrophils, timely release of *A. phagocytophilum* from infected cells is critical for its survival and spreading to neighboring cells once it develops into mature, infectious progenies. In human neutrophils, cortical F-actin is a long cross-linked peripheral meshwork that lies immediately beneath the plasma membrane [91,92], which serves as barrier for vesicle secretion/exocytosis [52]. Our study demonstrated that focal cortical F-actin depolymerization occurred at the cell periphery where mature *A. phagocytophilum*-containing vacuoles are in the process of spontaneous release, suggesting spatiotemporal regulation of actin dynamics for *A. phagocytophilum* intracellular retention and release. Furthermore, pharmacological depolymerization of actin cytoskeleton induced massive release of bacteria, indicating F-actin network serves as barrier for extracellular release of bacteria.

*A. phagocytophilum* undergoes a biphasic developmental cycle that transitions between an infectious DC form and a non-infectious replicative RC form [18,21], therefore, its intracellular development and extracellular release require tight coordination and regulation. *A. phagocytophilum* takes 1–2 days to fully mature into DC forms after infecting naïve host cells and subsequently released to extracellular spaces for cell-to-cell spreading [19]. As a result, the reduced infectivity of released bacteria following F-actin disruption at 1 dpi is likely due to the premature release of RC forms. Furthermore, during intracellular development of *A. phagocytophilum* in host cells, genes encoding T4SS system like *virB9* are up-regulated in the proliferating RC forms but down-regulated when *A. phagocytophilum* matures into DC forms [19], thus consequently reduced secreted T4SS effectors like AnkA in host cytosol. Since secreted T4SS effector proteins play essential roles for the intravacuolar bacterium like *Anaplasma* to hijack host cellular machinery including remodeling of actin cytoskeleton and regulation of secretory pathways [93,94], lack of the T4SS effector AnkA at DC forms of *A. phagocytophilum* likely caused deregulation of host cellular machinery, resulting in bacterial release.

As proposed in S8 Fig, the current study demonstrated that AnkA, the T4SS effector secreted at RC stage, can interact with host Actn4, gelsolin, and actin, and regulate the release of *A. phagocytophilum* from infected host cells. Actn4 is involved in binding F-actin to the plasma membrane [67], and gelsolin is an actin binding protein that mediates the rapid remodeling of actin filaments through severing, capping, and nucleating activities, especially in stress fiber-dependent cell functions [66,70,95,96]. Therefore, it’s possible that the interaction of Actn4 with AnkA-N could potentially induce its localization beneath the plasma membrane and enhance the polymerization of cortical F-actin to form extensive ruffling and blebbing structures on the plasma membrane; whereas the interaction of AnkA-C with gelsolin and actin might bridge cytosolic gelsolin-bound G- or F-actins to Actn4-bound F-actins at the cell periphery, thus enhancing cortical F-actin assembly and preventing the premature release of *A. phagocytophilum* from host cells. In addition, studies showed that gelsolin activity and perturbed actin dynamics could control efficient early HIV-1 infection [97]. Therefore, Actn4-gelsolin-actin system may be involved in host defense by promoting early extrusion of intracellular bacteria, and *A. phagocytophilum* subverts this innate immune response by AnkA to allow full maturation of infectious *A. phagocytophilum*. To our knowledge, AnkA is the first example of bacterial molecules that interact with gelsolin and Actn4.

Our study demonstrated that actin cytoskeleton and actin-based motility, which play critical roles in intracellular vesicular trafficking and regulated exocytosis [98,99], are also required for *A. phagocytophilum* internalization into the host cells and intracellular proliferation. Therefore, knockdown of ACTN4/Gelsolin might also affect bacterial invasion and infection, causing released bacteria remaining attached on cell surface but unable to reinfect the host cells. In addition, it is possible that AnkA also regulates intracellular bacterial growth through interaction with actin and actin-regulatory proteins. Nevertheless, further detailed investigations are needed to understand the regulation of *Anaplasma* release by AnkA or other bacterial proteins.

Most intracellular bacterial pathogens can exploit host cytoskeleton for their entry, intracellular motility, and cell-to-cell spreading, thereby facilitating infection [100–103]. For intra-cytosolic pathogens like *Rickettsia*, *Listeria*, *Burkholderia*, and *Shigella*, actin-based motility is critical for intracellular survival and intercellular spreading [103–105]. Several bacterial surface proteins or secreted effector proteins introduced into the host cytosol by specialized secretion systems, have been identified in regulating actin cytoskeleton dynamics [104–106]. For example, *Rickettsia* Sca2 can interact with actin directly [103,107], whereas others can indirectly alter actin rearrangements by mimicking actin nucleation-promoting factors like Cdc42, N-WASP, or Arp2/3, including *Shigella* autotransporter IcsA [108,109], *Listeria* ActA [110], *Burkholderia thailandensis* autotransporter BimA [111,112], or the spotted fever group *Rickettsia* WASP-like protein RickA [113]. For intravacuolar pathogens, *Salmonella* effector protein SipC and *Chlamydia* effector Tarp manipulate the host cell’s actin cytoskeleton for bacterial entry and replication [114–117], and *Legionella* VipA displays actin nucleation activities and alters host organelle trafficking [118].

However, for obligatory intravacuolar bacteria like *Anaplasma*, *Ehrlichia*, *Chlamydia*, and *Coxiella*, other than bacterial entry, roles and regulation of actin dynamics in intracellular infection remain mostly unknown [102,114,116,118–121]. Particularly knowledge on release mechanisms of these bacteria is limited. Furthermore, homologs of these bacterial actin-interacting effector proteins characterized above were not found in *A. phagocytophilum* genome. Studies have shown that host cell exit of *Chlamydia trachomatis* involves extrusion to release the whole inclusion containing bacteria, by actin polymerization via Rho GTPases, N-WASP, and the myosin-activating machinery recruited by a bacterial protein [119,122,123]. Interestingly, *Ehrlichia chaffeensis-*containing vacuoles were transported to the leading edge of filopodia of macrophages for intercellular transmission during the early stages of infection, likely involves N-WASP-dependent actin polymerization [82,124]. Recent studies proposed roles of multivesicular bodies for *A. phagocytophilum* release [23]; however, bacterial effectors potentially involved were not identified, and whether exocytosis of multivesicular body is linked to actin dynamics is unknown.

Our results suggested the critical roles of actin dynamics manipulated by AnkA in *A. phagocytophilum* release. Ankyrin repeat is one of the most common motifs that mediates protein-protein interactions [125]. Recent studies have identified three to four ankyrin repeat-containing proteins in *Anaplasma* and related *Ehrlichia* spp. [126,127], as well as many Ank-containing proteins of intracellular pathogens, such as *Wolbachia*, *Rickettsia*, *Orientia*, *Legionella*, and *Coxiella* spp. [125,127–129]. For example, *Coxiella burnetii* Ank proteins localize to a variety of subcellular regions in mammalian cells including microtubules, mitochondria, and the bacteria-containing vacuole membrane [130]; whereas *Legionella pneumophila* AnkN/AnkX protein prevents microtubule-dependent vesicular transport and interferes with fusion of the bacteria-containing vacuole with late endosomes in macrophages [131,132]. The Scrub typhus pathogen, *Orientia tsutsugamushi*, encodes remarkably large numbers of Ank-repeat proteins (47 in Ikeda strain, 37 in Boryong strain) [125], most of which target the endoplasmic reticulum or Golgi apparatus to regulate host cell secretory pathway [133,134]. Many of these Ank proteins are delivered into eukaryotic cells through type I or IV secretion system and involved in the regulation of host functions and disease development [135], suggesting a common theme evolved in these intracellular bacteria to subvert host cell functions with Ank-containing proteins.

Studies have demonstrated that host actin plays various roles in *A. phagocytophilum* infection. *A. phagocytophilum* induces tyrosine-phosphorylation of actin in tick vector *Ixodes scapularis* to regulate a gene crucial for *A. phagocytophilum* infection in tick cells [136], and the bacterium can secrete a T4SS effector AFAP, which interacts with host nucleolin to promote neutrophil cell adhesion [38]. The current study unraveled a new paradigm of microbial exploitation of actin dynamics and reveals a novel release mechanism regulated by a bacterial T4SS effector. The understanding of HGA pathogenesis involving the active exploitation of host actin dynamics will provide novel insights into bacterial survival and spreading strategies in general and could lead to novel therapeutic and preventive strategies for Anaplasmosis and related tick-borne zoonoses.

## Materials and methods

### Reagents and antibodies

Chemical inhibitors of cytoskeleton disruption (cytochalasin D, latrunculin B, C3-transferase, blebbistatin, wiskostatin, ML-7, Taxol, and nocodazole) were purchased from EMD Biosciences (San Diego, CA), and Gleevec was kindly provided by Norvatis (Basel, Switzerland) (Table 1) [33].

The mouse monoclonal antibody (mAb) 5C11 recognizing the N-terminal conserved region of P44 of *A. phagocytophilum* was produced as described previously [62]. Rabbit anti-AnkA IgG was produced against C-terminus of AnkA (aa 1,143–1,162: QRGKLRPVKGGAPDSTKDKT) (Fig 6A) by Proteintech and purified as described [33]. Mouse anti-Gelsolin (Gsn) and rabbit anti-Actin and α-Actinin-4 (Actn4) were purchased from Sigma (Saint Louis, MO). Mouse anti-α-Tubulin antibody, rabbit anti-Abi-1 antibodies, unconjugated or agarose-conjugated mouse anti-phosphotyrosine mAb PY99, and agarose-conjugated normal mouse or rabbit IgG were purchased from Santa Cruz Biotech (Santa Cruz, CA). Horseradish peroxidase (HRP)-conjugated anti-mouse or rabbit secondary antibodies were purchased from Cell Signaling Technology (Danvers, MA).

### Cultivation of *A. phagocytophilum* and host cells

*A. phagocytophilum* HZ strain was cultivated in human leukemia cell line HL-60 cells in RPMI 1640 medium [137]. Emerald green-expressing *A. phagocytophilum* HZ and mCherry-expressing HGE strains were obtained from Dr. Munderloh [79]. RF/6A cells were cultured in Advanced MEM and HEK293 cells were cultured in DMEM medium [138]. All culture media were supplemented with 8% fetal bovine serum (FBS) and 2% i-glutamine, and cells were maintained at 37°C in 5% CO_2_ and 95% air. No antibiotic was used throughout the study. To access the degree of bacterial infection in host cells, cells were centrifuged onto glass slides using a Shandon Cytospin 4 cytocentrifuge (Thermo Fisher, Kalamazoo, MI), and stained by Diff-Quik staining (Baxter Scientific Products, Obetz, OH).

Host cell-free *A. phagocytophilum* was purified from heavily infected HL-60 cells (>95% infectivity) by sonication, filtration through a 2.7-μm syringe filter to remove cell debris, and centrifugation at 10,000 × *g* for 10 min as previously described [138].

### Immunoprecipitation (IP), Western blot analysis, and protein identification by mass spectrometry

The whole cell lysates of *A. phagocytophilum*-infected or uninfected cells were prepared by lysis in modified RIPA (radioimmunoprecipitation) buffer (50 mM Tris-HCl, pH 7.4, 1% NP-40, 0.25% sodium deoxycholate, 150 mM NaCl, 1 mM EDTA) with freshly added protease and phosphatase inhibitor cocktails (EMD Biosciences). After incubation on ice for 20 min, cells were sonicated; and whole cell lysates were collected by centrifugation at 10,000 × g for 10 min.

For immunoprecipitation, whole cell lysates were incubated with agarose-conjugated anti-phosphotyrosine mAb (PY99), or Anti-AnkA IgG, then with protein A-agarose (Santa Cruz). Immunocomplexes were resuspended in 2× SDS sample buffer and boiled for 5 min. Samples were subjected to 8% SDS-PAGE, GelCode Blue staining according to manufacturer’s instructions (Thermo Fisher Scientific, Waltham, MA), and Western blot analysis as described previously [139]. Reacting bands were visualized with enhanced chemiluminescence and images were captured using an LAS3000 image documentation system (FUJIFILM Medical Systems USA, Stamford, CT). Band densities were quantitated by ImageQuaNT (Cytiva, Marlborough MA).

For protein identification with capillary-liquid chromatography-nanospray tandem mass spectrometry (Nano-LC/MS/MS), polyacrylamide gel was Coomassie blue-stained and tryptic digested. Mass spectrometry was performed at the Ohio State University (OSU) Mass Spectrometry and Proteomics Facility.

### Plasmids construction, recombinant protein purification, and GST-pull down assay

GST- or EGFP-tagged FL-, N-, or C-terminus of AnkA proteins were constructed by PCR amplification with specific primers (S1 Table) and ligation with target plasmids pET41a(+) (Novagen, San Diego, CA) or pEGFP-N1 (Clontech, Mountain View, CA), respectively. GST-tagged fusion proteins containing C-terminal 6×His-tag were expressed in *E. coli* strain BL21 (DE3) (Novagen) by 1 mM isopropyl β-D-1-thiogalactopyranoside induction for 4 h at 30°C as described previously [140,141]. Briefly, *E. coli* was lysed by sonication in binding/lysis buffer (50 mM sodium phosphate, pH 8.0, 0.3 M NaCl, 10 mM imidazole). Recombinant proteins were affinity purified from soluble fractions with a HIS-Select Cartridge (Sigma), and dialyzed against Dulbecco’s modified phosphate-buffered saline (PBS: 8 mM Na_2_HPO_4_, 1.47 mM KH_2_PO_4_, 2.67 mM KCl, 137.9 mM NaCl, pH 7.4).

For GST-pull down assay, recombinant GST-fusion proteins (20 µg) were immobilized on 20 µl of glutathione-sepharose beads (GE Health Sciences, Piscataway, NJ) and mixed with 500 µl of HL-60 cell lysates in RIPA buffer for 2 h. The beads were washed extensively with PBS buffer, and bound proteins were eluted by glutathione-elution buffer. Samples were separated by SDS-PAGE, followed by GelCode Blue staining or Western blot analysis.

### Pyrene-actin polymerization assay

The effects of AnkA on actin kinetics were measured by *in vitro* pyrene-actin polymerization assay according to the manufacturer’s instructions (Cytoskeleton, Denver, CO) [71,72]. Briefly, pyrene-labeled actin monomers (0.4 mg/ml) are prepared in G-buffer (5 mM Tris, pH 8.0, 0.2 mM CaCl_2_, 0.2 mM ATP), and centrifuged to remove actin polymers. Recombinant GST-AnkA fusion proteins (FL-, N-, or C-terminus) were normalized to 20 µM in Tris buffer (5 mM Tris-HCl, pH 7.5). Approximately 1 µg of pyrene-actin was gently mixed with 1 µM of GST-fusion proteins and 1/10 dilution of HL-60 cell lysate that served as host actin-nucleation promoting factors in a volume of 200 µl Tris buffer for 10 min [142]. Actin polymerization was initiated by the addition of actin polymerization buffer (50 mM KCl, 2 mM MgCl_2_, and 1 mM ATP). The reaction was monitored at 25°C for 60 min in a Gemini XS Spectrofluorometer (Molecular Devices, Sunnyvale, CA) to measure the fluorescence emissions with an excitation wavelength (Ex) at 365 nm and an emission wavelength (Em) at 407 nm. Data were analyzed and plotted by Microsoft Excel (Microsoft, Redmond, WA).

### Transfection, immunofluorescence assay, and fluorescence microscopy

Plasmids encoding pEGFP-AnkA proteins were purified using EndoFree Maxi plasmid kit (Qiagen, Valencia, CA) and transfected into RF/6A cells by Fugene HD reagent (Promega, Madison, WI). At 2 d post transfection, cells were fixed in 4% paraformaldehyde at room temperature for 15 min, and permeabilized with PGS buffer (PBS supplemented with 0.1% gelatin and 0.3% saponin) for 15 min. F-actin was labeled with AF647-conjugated phalloidin (Invitrogen, Carlsbad, CA) for 30 min.

For antibody labeling, *A. phagocytophilum*-infected HL-60 cells at 1 or 2 d pi following inhibitor treatment were cytocentrifuged onto glass slides, fixed and permeabilized as described above. Cells were labeled with mouse anti-*A. phagocytophilum* P44 mAb and rabbit anti-AnkA IgG, then with AF488–conjugated goat anti-mouse and AF555–conjugated goat anti-rabbit secondary IgGs (Invitrogen) in blocking buffer (DPBS containing 1% bovine serum albumin) for 1 h each. Fluorescence and differential interference contrast (DIC) images were captured and analyzed using a DeltaVision deconvolution microscope (Applied Precision, Issaquah, WA), or an Olympus FlowView 1000 Laser Scanning Confocal microscopy (Olympus, Center Valley, PA). Line profile analysis of fluorescence intensities was performed using FIJI ImageJ2 [143].

For flow cytometry analysis, *A. phagocytophilum*-infected or uninfected HL-60 cells at 1 d and 2 d pi (1.5 × 10^6^ cells) were washed with PBS, fixed in 4% PFA for 20 min, and labeled with AF647-Phalloidin (5 µl diluted in 200 µl PGS) for 30 min. Fluorescence–labeled samples were detected by an Accuri C6 flow cytometer according to the manufacturer’s instructions (BD Biosciences, San Jose, CA).

### Viability assays of bacteria and host cells

Viability of *A. phagocytophilum* and host cells were assessed using membrane-permeability based LIVE/DEAD *Bac*Light Bacterial Viability Kits (Invitrogen) according to the manufacturer’s instructions. Briefly, HL-60 cells infected with *A. phagocytophilum* were treated with 10 µM cytochalasin D or DMSO solvent control at 1 d pi for 18 h, or at 2 d pi for 5 h. Cells were resuspended in PBS, plated into each well of a 96-well plate, and incubated for 15 min at dark with mixtures of the SYTO 9 green-fluorescent nucleic acid stain and the red-fluorescent nucleic acid stain propidium iodide (PI). An aliquot of cells was centrifuged onto slides and observed under DeltaVision fluorescence microscope immediately. Fluorescence emissions of SYTO 9 (green, Em_510 nm_) and PI (red, Em_630 nm_) were measured under Ex_470 nm_ in a Gemini XS Spectrofluorometer, and the green/red fluorescence ratios were calculated for the relative proportions of live/dead bacteria or cells.

Alternatively, treated host cells were subjected to metabolic activity-based CyQUANT MTT (3-[4,5-dimethylthiazol-2-yl]-2,5 diphenyl tetrazolium bromide) Cell Viability Assay (Thermo Fisher). Briefly, 1×10^5^ cells in 100 μL culture medium per well were seeded in a 96-well plate and incubated with 10 μL of the 12-mM MTT for at 37°C for 4 hours. The plate was centrifuged to sediment the cells, and all but 25 μL of medium was removed from the wells. 50 μL of DMSO (as a solubilizing agent for formazan) was added to each well and mixed thoroughly. After incubation at 37°C for 10 minutes, the absorbance was measured at 540 nm.

### Transmission and scanning electron microscopy (TEM & SEM)

For TEM sample preparation [144], *A. phagocytophilum*-infected HL-60 cells following cytochalasin D treatment were fixed in 3% glutaraldehyde (EM Sciences, Hatfield, PA), 2% formaldehyde, and 0.02% trinitrophenol, and stained at 4°C in reduced osmium tetraoxide. After uranyl acetate block staining, samples were dehydrated with graded series of ethanol. Ultra-thin sections (60 nm) were stained with uranyl acetate and lead citrate, and observed under a Philips EM 300 transmission EM (Philips, Hillsboro, OR) at 60 kV.

For SEM, infected cells following cytochalasin D treatment were fixed with 2.5% glutaraldehyde, dehydrated with ethanol, and subjected to critical point drying using hexamethyldisilazane (Ted Pella, Redding, CA). Dried samples were mounted on an aluminum stub, coated with a very thin film of gold and palladium, and observed under a FEI NOVA nanoSEM 400 scanning EM (FEI, Hillsboro, OR) at OSU Campus Microscopy and Imaging Facility (CMIF).

### Lentiviral-shRNAs (short-hairpin RNAs) transduction of HL-60 cells

Validated shRNA-expressing lentiviral particles that target human Actn4 (GenBank# NM_004924) or gelsolin (GenBank# NM_0001777) were purchased from Sigma. Non-mammalian shRNA lentiviral particles were used as transduction control (SHC002V, Sigma). HL-60 cells were transduced with lentiviral particles at multiplicity of infection (MOI) of 1–10 (50 µl × 10^6^ TU/ml for 5 × 10^4^ cells in 0.5 ml RPMI medium) in the presence of 8 µg/ml of polybrene (Sigma). Positive-transduced HL-60 cells expressing shRNAs were selected with 2 µg/ml puromycin at 3 d post transduction for more than 3 d. Stable cell lines with gene silencing were infected with host cell-free *A. phagocytophilum* (MOI of 50). At 2 dpi, infection level was examined by Diff-Quik staining and cell lysates were subjected to Western blot analysis.

### Reverse transcription and quantitative polymerase chain reaction (RT-qPCR) analysis

A. phagocytophilum–infected HL-60 cells were pelleted by centrifugation at 400 × *g* for 5 min, and the culture supernatant containing released bacteria were further harvested by high-speed centrifugation at 10,000 × *g* for 10 min. DNA or RNA samples were purified using the QIAamp DNA blood mini kit or RNeasy Plus mini kit, respectively (Qiagen, Valencia, CA). Total RNA (2 µg) was reverse transcribed using SuperScript III reverse transcriptase (Invitrogen) and Random Decamers primers (Invitrogen). Quantitative PCR (20 µl total volume) was performed with 1 µl of cDNA (corresponding to 0.2–0.4 µg of total DNA or RNA) and 0.25 µM primers targeting A. phagocytophilum 16S rRNA gene and human/monkey glyceraldehyde 3-phosphate dehydrogenase (GAPDH) gene (S1 Table) [35,145], using a Maxima SYBR green/ROX qPCR Master Mix (Thermo Fisher) according to the manufacturer’s protocol in an MX3000P (Stratagene, La Jolla, CA) or the AriaMx Real-Time PCR System (Agilent, Santa Clara, CA).

## Supporting information

S1 Movie Spatiotemporal cortical F-actin depolymerization at the site of spontaneous *A. phagocytophilum* release. Emerald green-expressing *A. phagocytophilum* HZ (Emerald-HZ)-infected HL-60 cells at 2 dpi were fixed, incubated with AF647-conjugated phalloidin to label F-actin (pseudo-colored red), and observed under DeltaVision Microscope. Images were captured with *Z*-stack steps at 0.15 µm from the top to the bottom of the cells, and three-dimensional reconstruction of fluorescence microscopy images (as shown in Fig 1B) along the *Z*-stacks following DeltaVision deconvolution processing were performed by SoftWoRx software. Bar, 15 µm.
(MOV)

S1 Fig Viability assay of *A. phagocytophilum* and infected host HL-60 cells following F-actin disruption. *A. phagocytophilum*-infected HL-60 cells were treated with 10 µM cytochalasin D (Cyt. D), 1 µM latrunculin B (LaB), or DMSO solvent control (CTL) at 1 dpi for 18 h, or at 2 dpi for 5 h. (**A-B**) Cells were washed, resuspended in PBS, then incubated with SYTO 9 and propidium iodide (PI) for 15 min at dark. (**A**) An aliquot of cells was centrifuged onto slides and observed under fluorescence microscope immediately. Green color indicates viable cells/bacteria, while red staining indicates dead organisms (mostly undetectable). Bar, 10 µm. (**B**) Fluorescence emissions of SYTO 9 (green, Em_510nm_) and PI (red, Em_630nm_) for 18 h treatment groups were measured under Ex_470nm_ in a Gemini XS Spectrofluorometer, and the ratios of green/red fluorescence intensities were calculated. Data were presented as the mean ± SD from two independent experiments with triplicates; ns, not significant by Student’s *t* test. (**C**) Alternatively, treated cells were subjected to metabolic activity-based CyQUANT MTT Cell Viability Assay. Briefly, 1×10^5^ cells in 100 μL culture medium per well were seeded in a 96-well plate and incubated with 10 μL of the 12-mM MTT for at 37°C for 4 h. Insoluble formazan converted from MTT in viable cells were solubilized by DMSO, and the absorbance was measured at 540 nm. ns, not significant by ANOVA. Data were representative of two independent experiments and presented as the mean ± SD of triplicate samples.
(TIF)

S2 Fig F-actin disruption induced the release of *A. phagocytophilum* from infected host cells. (**A**) *A. phagocytophilum*-infected HL-60 cells at 1–2 dpi were treated with 1 µM latrunculin B (LaB) or DMSO solvent control for the indicated time points (e.g., 43 hpi + 5 h LaB treatment, or 24 h pi + 24 h LaB treatment). At 48 hpi, cells were cytospun onto slides for Diff-Quik staining. White arrows, *Anaplasma*-containing vacuoles in the process of exocytosis; Black arrows, released bacteria remaining associated with infected host cells. Bar, 10 µm. Images were representative data from three independent experiments with similar results. (**B**) Numbers of *A. phagocytophilum* inside HL-60 cells (intracellular), or individual bacteria either present at extracellular spaces or remain attached to the cell surface (released) were quantified by counting approximately 80–100 cells from two independent experiments. * *P* < 0.05; ** *P* < 0.01; **** *P* < 0.0001: significant difference by nested one-way ANOVA (numbers of released or intracellular *A. phagocytophilum* of LaB vs. control groups). (**C**) *A. phagocytophilum*-infected HL-60 cells at 2 dpi (~90% infectivity) were treated with 10 µM cytochalasin D for 2.5 h (as shown in Fig 2C), and then cytospun onto slides for Diff-Quik staining. Numbers of *A. phagocytophilum* inside HL-60 cells (Intracellular *Ap*), or individual bacteria either present at extracellular spaces or remain attached to the cell surface (Released *Ap*) were quantitated by counting approximately 80–100 cells from two independent experiments. ** *P* < 0.01, significant difference by one-way ANOVA.
(TIF)

S3 Fig Microtubule disruption by nocodazole did not induce *A. phagocytophilum* release. (**A**) *A. phagocytophilum*-infected HL-60 cells at 1–2 dpi were treated with 10 µM nocodazole (Noc) or DMSO solvent control for the indicated time points (e.g., 43 hpi + 5 h Noc treatment, or 30 hpi + 18 h Noc treatment). At 48 hpi, cells were cytospun onto slides for Diff-Quik staining. Images were representative data from at least 3 independent experiments with similar results. Bar, 10 µm. (**B**) Numbers of *A. phagocytophilum* inside HL-60 cells (intracellular), or individual bacteria either present at extracellular spaces or remain attached to the cell surface (released) were quantitated by counting approximately 80–100 cells from two independent experiments. No significant difference by one-way ANOVA between control and Noc treatment.
(TIF)

S4 Fig Three-dimensional structure prediction of AnkA domains and interaction with actin and actin-regulatory proteins. Three-dimensional protein structures of *A. phagocytophilum* AnkA and protein-protein interactions were predicted by AlphaFold 3 server (https://alphafoldserver.com). (**A**) 3D structure of AnkA, showing N-terminal domains (44–859 aa) containing mostly ankyrin-repeats that are characterized by helix-loop-helix motifs. C-terminus contains mostly intrinsically disordered regions except for a short α-helix motif (1,170 ~ 1,190 aa). Numbers indicated amino acids positions. Color-coded protein strands: red, α-helix; yellow (Y1–Y6), tyrosine phosphorylation motifs. (**B-D**) Interaction of AnkA with human α-Actinin 4 (ACTN4, **B**), Gelsolin (GSN, **C**), or β-Actin (ACTB, **D**). Pink, AnkA; Blue, human proteins; green box, predicted AnkA motifs interacting with ACTN4 (5–192 aa, **B**), Gelsolin (1,169–1,192, **C**), and ACTB (1,133–1,144 aa, **D**). Protein length and NCBI accession numbers: *A. phagocytophilum* AnkA (1,232 aa), WP_011450840.1; human α-Actinin 4 (911 aa), NP_004915.2; β-Actin (375 aa), NP_001092.1; Gelsolin (731 aa), NP_937895.1.
(TIF)

S5 Fig Line profiling analysis of fluorescence intensities and colocalization of EGFP-AnkA with F-Actin. RF/6A cells were transfected with plasmids encoding EGFP or EGFP-tagged AnkA proteins using Fugene HD reagent for 2 d, and labeled with AF647-phalloidin (pseudo-colored red) as in Fig 7. Fluorescence images were captured using a DeltaVision deconvolution microscope system. Line profile analyses were performed using FIJI ImageJ2 to determine fluorescence intensities of EGFP or EGFP-AnkA (green lines) and AF647-phalloidin (red lines) along the yellow lines for the merged images in Fig 7. Open arrows, cortical F-actin at the cell edge; solid arrows, F-actin stress fibers in the cytosol. Bar, 10 µm.
(TIF)

S6 Fig Ectopically expressed AnkA colocalizes with stress fibers or cortical F-actin in *A. phagocytophilum-*infected RF/6A cells. RF/6A cells were transfected with plasmids encoding EGFP or EGFP-tagged AnkA proteins using Fugene HD reagent for 1 d and infected with mCherry-expressing *A. phagocytophilum* HGE strain (mCherry-HGE). At 1 dpi (2 dpt), cells were fixed, labeled with AF647-phalloidin, and examined under DeltaVision deconvolution microscope. AF647-phalloidin labeling of F-actin was pseudo-colored grey in individual channels, or blue in merged channels. Right panels, 3× enlargement of box areas in merged images; DIC, Differential interference contrast. Arrows indicate colocalization of EGFP-AnkA with F-actin. Bar, 10 µm.
(TIF)

S7 Fig Effects of cytoskeleton-disrupting agents on internalization and proliferation of *A. phagocytophilum* in host cells. (**A**) To examine effects of cytoskeleton-disruption on bacterial internalization, naïve HL-60 cells were pretreated with inhibitors for 2 h and infected with host cell-free *A. phagocytophilum* (*Ap*) for 4 h in the presence of inhibitors. Cells were washed to remove inhibitors as well as unbound or uninternalized bacteria. (**B**–**D**) Alternatively, *A. phagocytophilum*-infected HL-60 were treated with inhibitors or DMSO solvent control (CTL) at 1 d pi for 16 h to determine effects of inhibitors on bacterial proliferation and release. Inhibitors and abbreviations: F-actin polymerization (cytochalasin D, CytD; latrunculin B, LaB); Abl-1 kinase (Gleevec), Rho GTPase (C3-transferase, C3T), Myosin II (blebbistatin, Bleb), MLCK (ML-7), N-WASP (Wiskostatin, Wisk), or microtubules (nocodazole, Noc; Taxol). Cells were cytospun onto slides for Diff-Quik staining, and infectivities were examined by counting numbers of intracellular *Ap* organisms in 100 cells in triplicates (**A-B**). Released *Ap* organisms either at extracellular spaces or attached to the cell surface (**C**) were quantified by counting approximately 80–100 cells. (**A-C**) Data were presented as the mean ± SD from three independent experiments. * *P* < 0.05; ** *P* < 0.01; **** *P* < 0.0001; ns, not significant; compared to the CTL group by one-way ANOVA. (**D**) Representative Diff-Quik staining images of panel **B**. Bar, 10 µm.
(TIF)

S8 Fig Proposed model of spatiotemporal regulation of cortical actin dynamics by AnkA for *A. phagocytophilum* intracellular retention and release. In proliferating (RC form)-stage of *A. phagocytophilum*-infected host cells, AnkA is secreted into host cells and interacts with Actn4, gelsolin, and actin at cell periphery, where the cortical F-actin network prevents the release of premature RC forms of *A. phagocytophilum* from host cells. The interaction of AnkA with host Abi-1 and Abl-1 tyrosine kinase, and subsequent AnkA phosphorylation are required for peripheral localization of AnkA and its interaction with F-actin. Mature DC forms of *A. phagocytophilum*, which turn down the expression of T4S apparatus and effector proteins including VirB9 and AnkA, could cause localized F-actin disassembly at cell periphery, thus inducing the release of mature *A. phagocytophilum* from infected cells. Illustrated by Tim Vojt.
(TIF)

S1 Table Primer sequences for cloning of *A. phagocytophilum* AnkA protein and qPCR analysis. (PDF)

S1 Raw Data All numerical data values used in graphical forms. (XLSX)

S1 Raw Images Uncropped and unadjusted images for all gel stainings and Western blotting results. (PDF)
