# Supplementary figures and images for: Obligatory intracellular bacterium Anaplasma phagocytophilum AnkA regulates actin dynamics and spatiotemporal bacterial release
Source: PLoS Pathog. 2026 Jun 24;22(6):e1014350. doi: 10.1371/journal.ppat.1014350 (PMC13293403; doi:10.1371/journal.ppat.1014350)

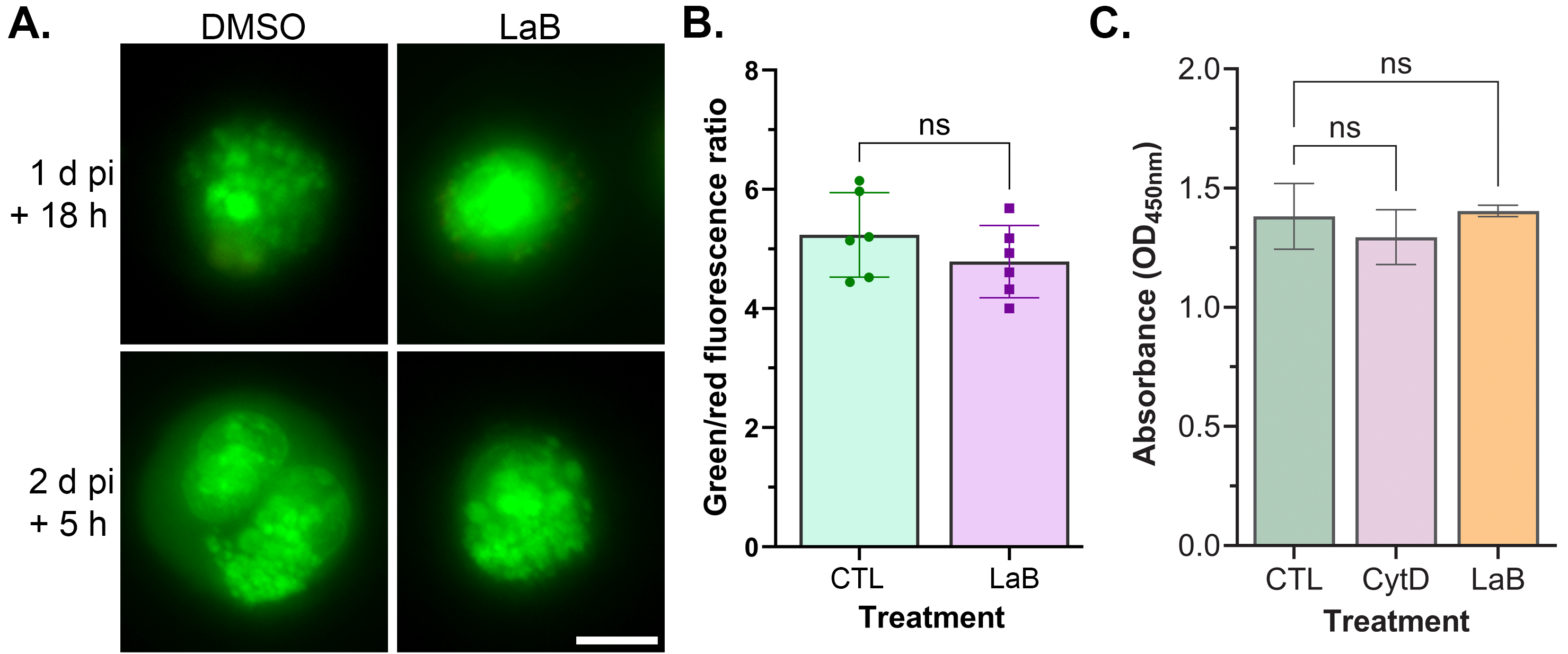

Supplement: S1 Fig — A. phagocytophilum-infected HL-60 cells were treated with 10 µM cytochalasin D (Cyt. D), 1 µM latrunculin B (LaB), or DMSO solvent control (CTL) at 1 dpi for 18 h, or at 2 dpi for 5 h. (A-B) Cells were washed, resuspended in PBS, then incubated with SYTO 9 and propidium iodide (PI) for 15 min at dark. (A) An aliquot of cells was centrifuged onto slides and observed under fluorescence microscope immediately. Green color indicates viable cells/bacteria, while red staining indicates dead organisms (mostly undetectable). Bar, 10 µm. (B) Fluorescence emissions of SYTO 9 (green, Em510nm) and PI (red, Em630nm) for 18 h treatment groups were measured under Ex470nm in a Gemini XS Spectrofluorometer, and the ratios of green/red fluorescence intensities were calculated. Data were presented as the mean ± SD from two independent experiments with triplicates; ns, not significant by Student’s t test. (C) Alternatively, treated cells were subjected to metabolic activity-based CyQUANT MTT Cell Viability Assay. Briefly, 1×10^5^ cells in 100 μL culture medium per well were seeded in a 96-well plate and incubated with 10 μL of the 12-mM MTT for at 37°C for 4 h. Insoluble formazan converted from MTT in viable cells were solubilized by DMSO, and the absorbance was measured at 540 nm. ns, not significant by ANOVA. Data were representative of two independent experiments and presented as the mean ± SD of triplicate samples. (TIF) [file ppat.1014350.s002.tif]

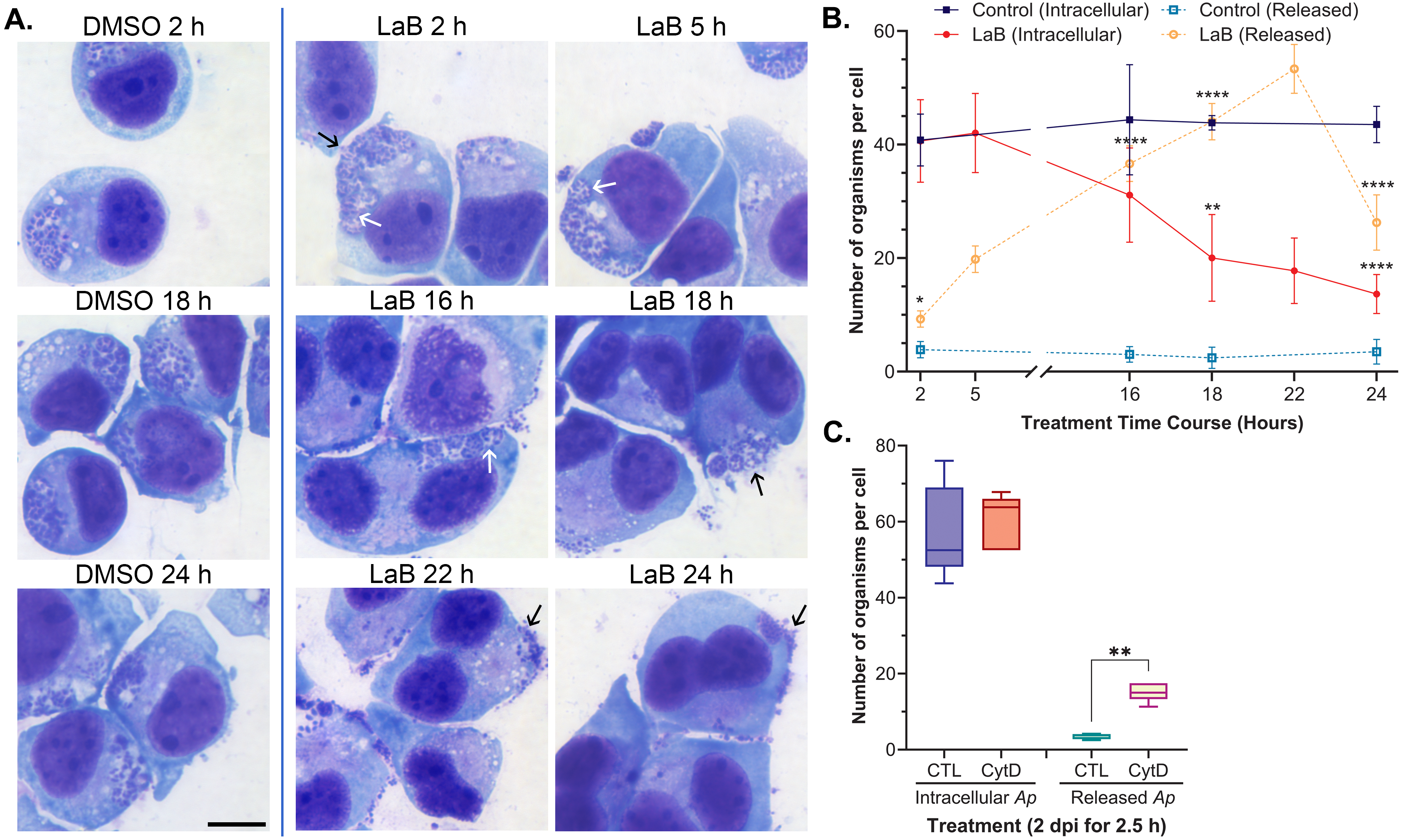

Supplement: S2 Fig — (A) A. phagocytophilum-infected HL-60 cells at 1–2 dpi were treated with 1 µM latrunculin B (LaB) or DMSO solvent control for the indicated time points (e.g., 43 hpi + 5 h LaB treatment, or 24 h pi + 24 h LaB treatment). At 48 hpi, cells were cytospun onto slides for Diff-Quik staining. White arrows, Anaplasma-containing vacuoles in the process of exocytosis; Black arrows, released bacteria remaining associated with infected host cells. Bar, 10 µm. Images were representative data from three independent experiments with similar results. (B) Numbers of A. phagocytophilum inside HL-60 cells (intracellular), or individual bacteria either present at extracellular spaces or remain attached to the cell surface (released) were quantified by counting approximately 80–100 cells from two independent experiments. * P < 0.05; ** P < 0.01; **** P < 0.0001: significant difference by nested one-way ANOVA (numbers of released or intracellular A. phagocytophilum of LaB vs. control groups). (C) A. phagocytophilum-infected HL-60 cells at 2 dpi (~90% infectivity) were treated with 10 µM cytochalasin D for 2.5 h (as shown in Fig 2C), and then cytospun onto slides for Diff-Quik staining. Numbers of A. phagocytophilum inside HL-60 cells (Intracellular Ap), or individual bacteria either present at extracellular spaces or remain attached to the cell surface (Released Ap) were quantitated by counting approximately 80–100 cells from two independent experiments. ** P < 0.01, significant difference by one-way ANOVA. (TIF) [file ppat.1014350.s003.tif]

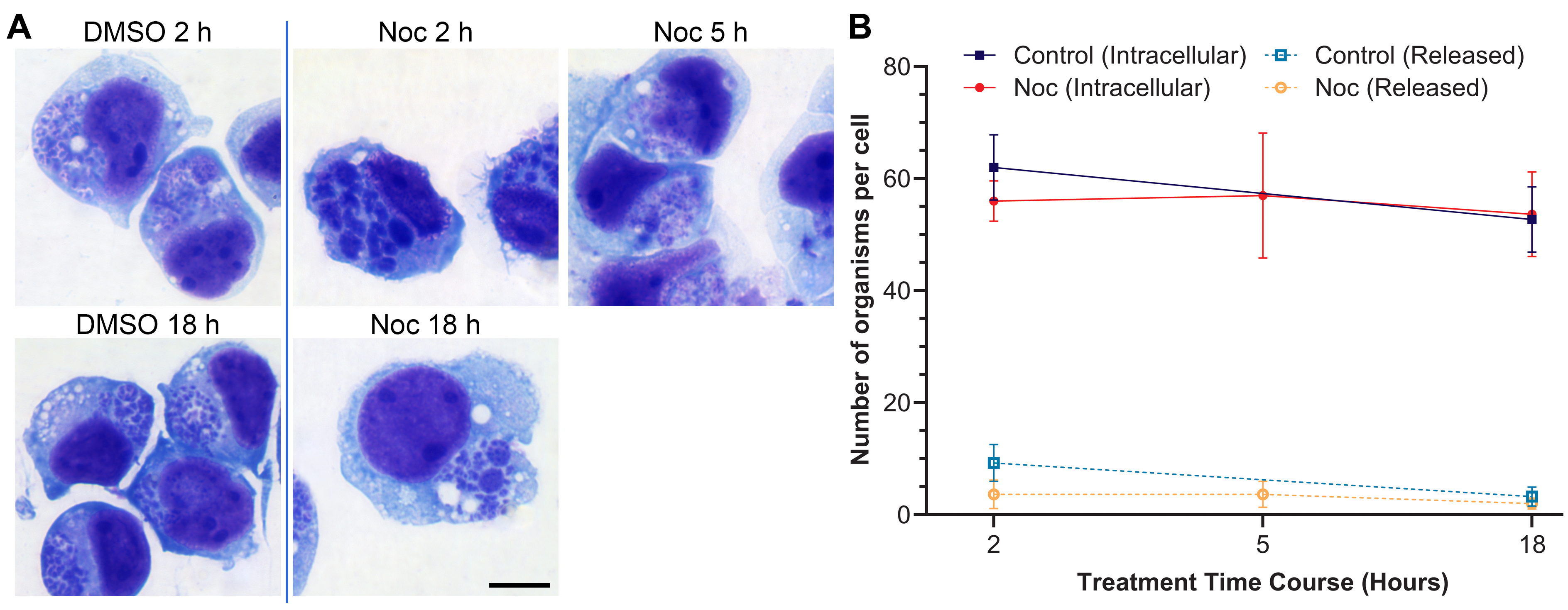

Supplement: S3 Fig — (A) A. phagocytophilum-infected HL-60 cells at 1–2 dpi were treated with 10 µM nocodazole (Noc) or DMSO solvent control for the indicated time points (e.g., 43 hpi + 5 h Noc treatment, or 30 hpi + 18 h Noc treatment). At 48 hpi, cells were cytospun onto slides for Diff-Quik staining. Images were representative data from at least 3 independent experiments with similar results. Bar, 10 µm. (B) Numbers of A. phagocytophilum inside HL-60 cells (intracellular), or individual bacteria either present at extracellular spaces or remain attached to the cell surface (released) were quantitated by counting approximately 80–100 cells from two independent experiments. No significant difference by one-way ANOVA between control and Noc treatment. (TIF) [file ppat.1014350.s004.tif]

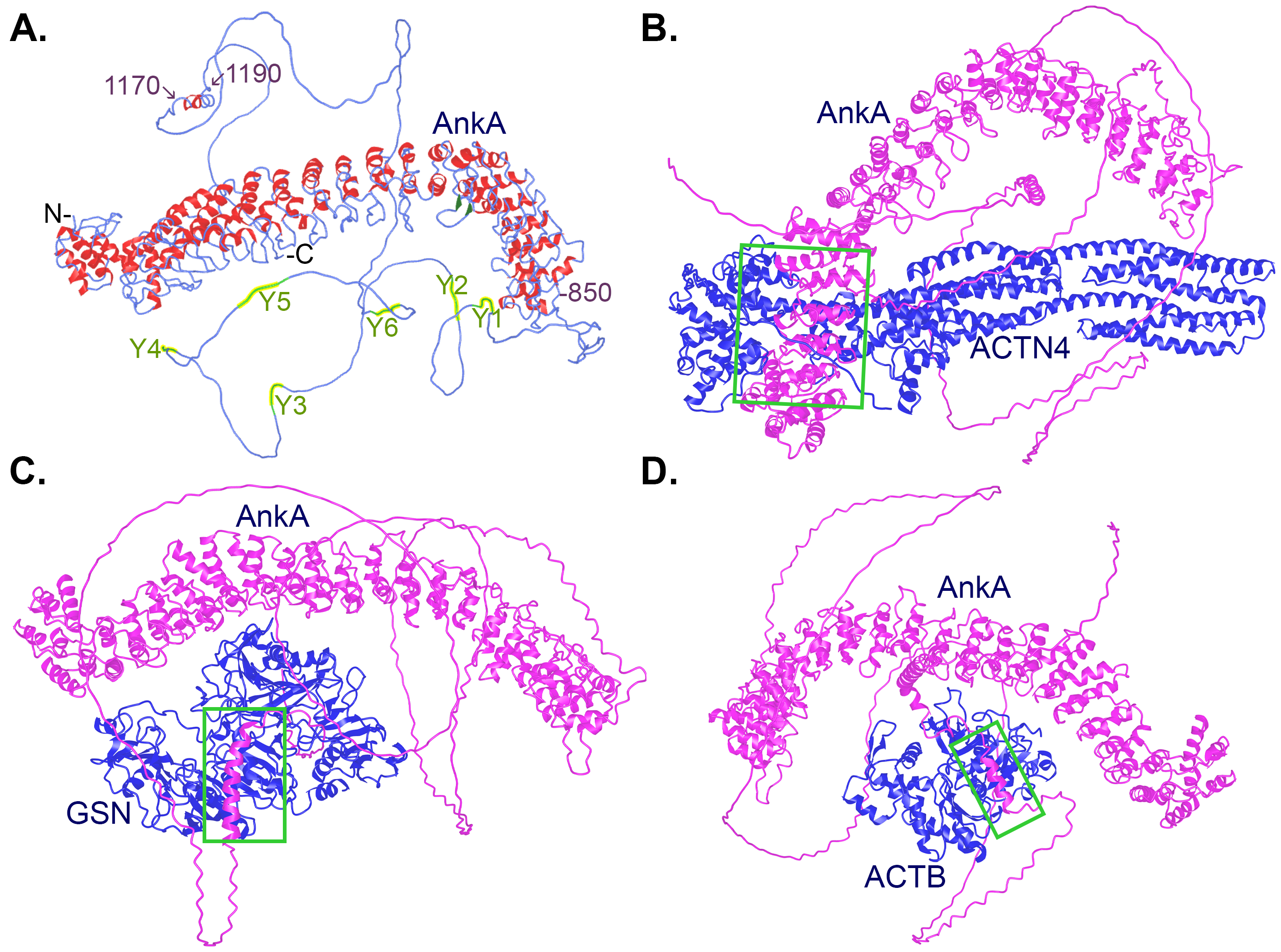

Supplement: S4 Fig — Three-dimensional protein structures of A. phagocytophilum AnkA and protein-protein interactions were predicted by AlphaFold 3 server (https://alphafoldserver.com). (A) 3D structure of AnkA, showing N-terminal domains (44–859 aa) containing mostly ankyrin-repeats that are characterized by helix-loop-helix motifs. C-terminus contains mostly intrinsically disordered regions except for a short α-helix motif (1,170 ~ 1,190 aa). Numbers indicated amino acids positions. Color-coded protein strands: red, α-helix; yellow (Y1–Y6), tyrosine phosphorylation motifs. (B-D) Interaction of AnkA with human α-Actinin 4 (ACTN4, B), Gelsolin (GSN, C), or β-Actin (ACTB, D). Pink, AnkA; Blue, human proteins; green box, predicted AnkA motifs interacting with ACTN4 (5–192 aa, B), Gelsolin (1,169–1,192, C), and ACTB (1,133–1,144 aa, D). Protein length and NCBI accession numbers: A. phagocytophilum AnkA (1,232 aa), WP_011450840.1; human α-Actinin 4 (911 aa), NP_004915.2; β-Actin (375 aa), NP_001092.1; Gelsolin (731 aa), NP_937895.1. (TIF) [file ppat.1014350.s005.tif]

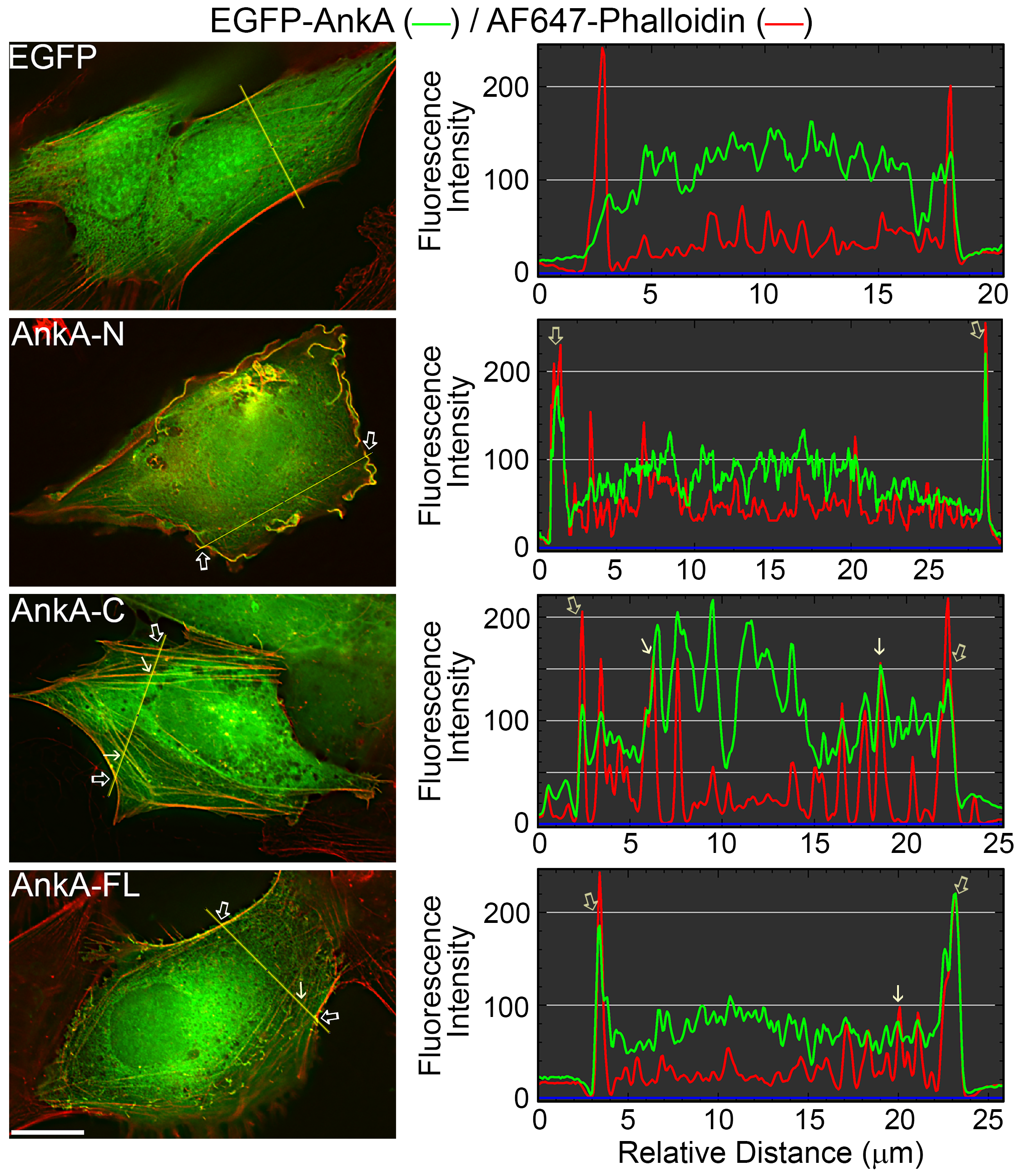

Supplement: S5 Fig — RF/6A cells were transfected with plasmids encoding EGFP or EGFP-tagged AnkA proteins using Fugene HD reagent for 2 d, and labeled with AF647-phalloidin (pseudo-colored red) as in Fig 7. Fluorescence images were captured using a DeltaVision deconvolution microscope system. Line profile analyses were performed using FIJI ImageJ2 to determine fluorescence intensities of EGFP or EGFP-AnkA (green lines) and AF647-phalloidin (red lines) along the yellow lines for the merged images in Fig 7. Open arrows, cortical F-actin at the cell edge; solid arrows, F-actin stress fibers in the cytosol. Bar, 10 µm. (TIF) [file ppat.1014350.s006.tif]

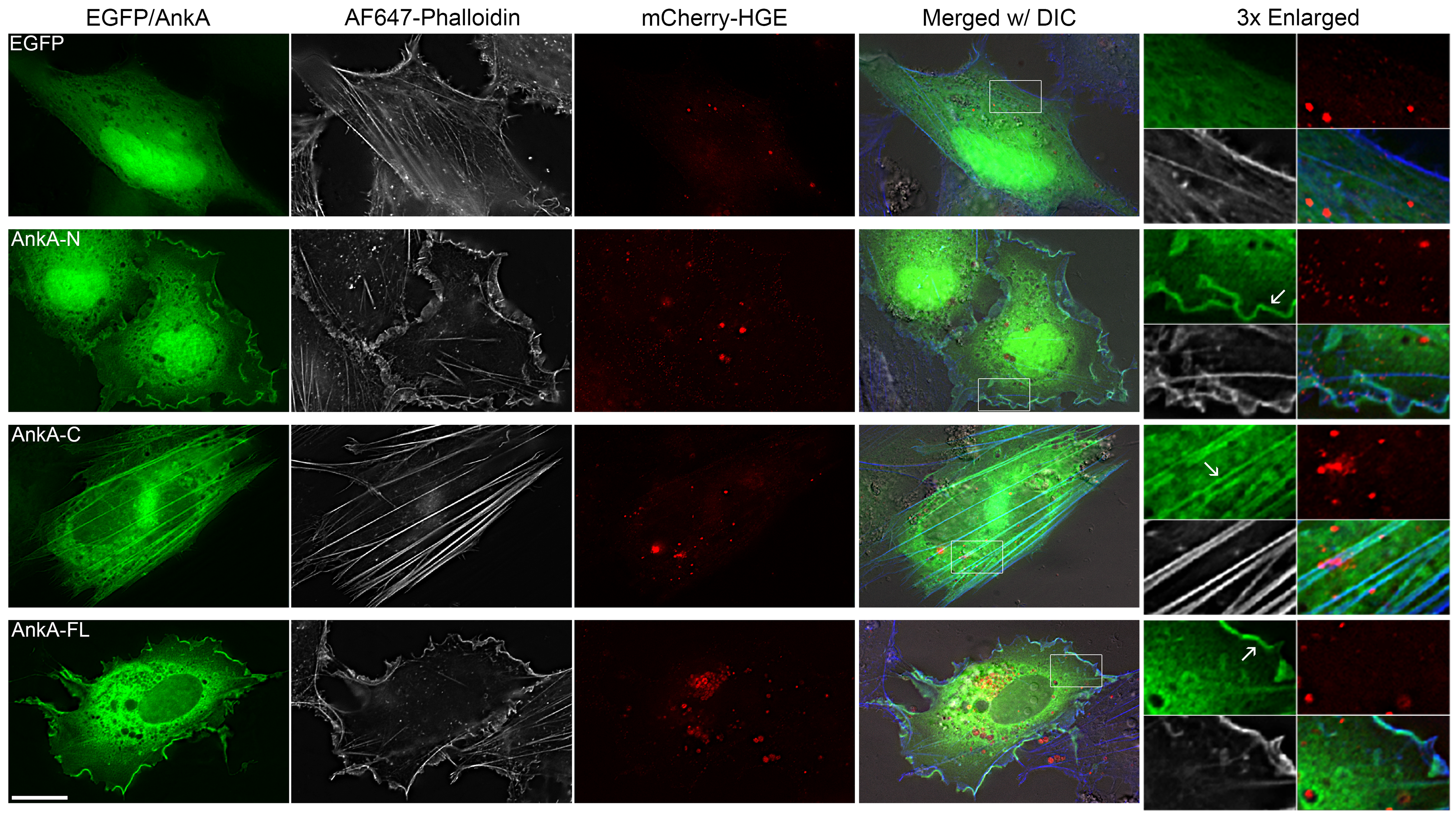

Supplement: S6 Fig — RF/6A cells were transfected with plasmids encoding EGFP or EGFP-tagged AnkA proteins using Fugene HD reagent for 1 d and infected with mCherry-expressing A. phagocytophilum HGE strain (mCherry-HGE). At 1 dpi (2 dpt), cells were fixed, labeled with AF647-phalloidin, and examined under DeltaVision deconvolution microscope. AF647-phalloidin labeling of F-actin was pseudo-colored grey in individual channels, or blue in merged channels. Right panels, 3× enlargement of box areas in merged images; DIC, Differential interference contrast. Arrows indicate colocalization of EGFP-AnkA with F-actin. Bar, 10 µm. (TIF) [file ppat.1014350.s007.tif]

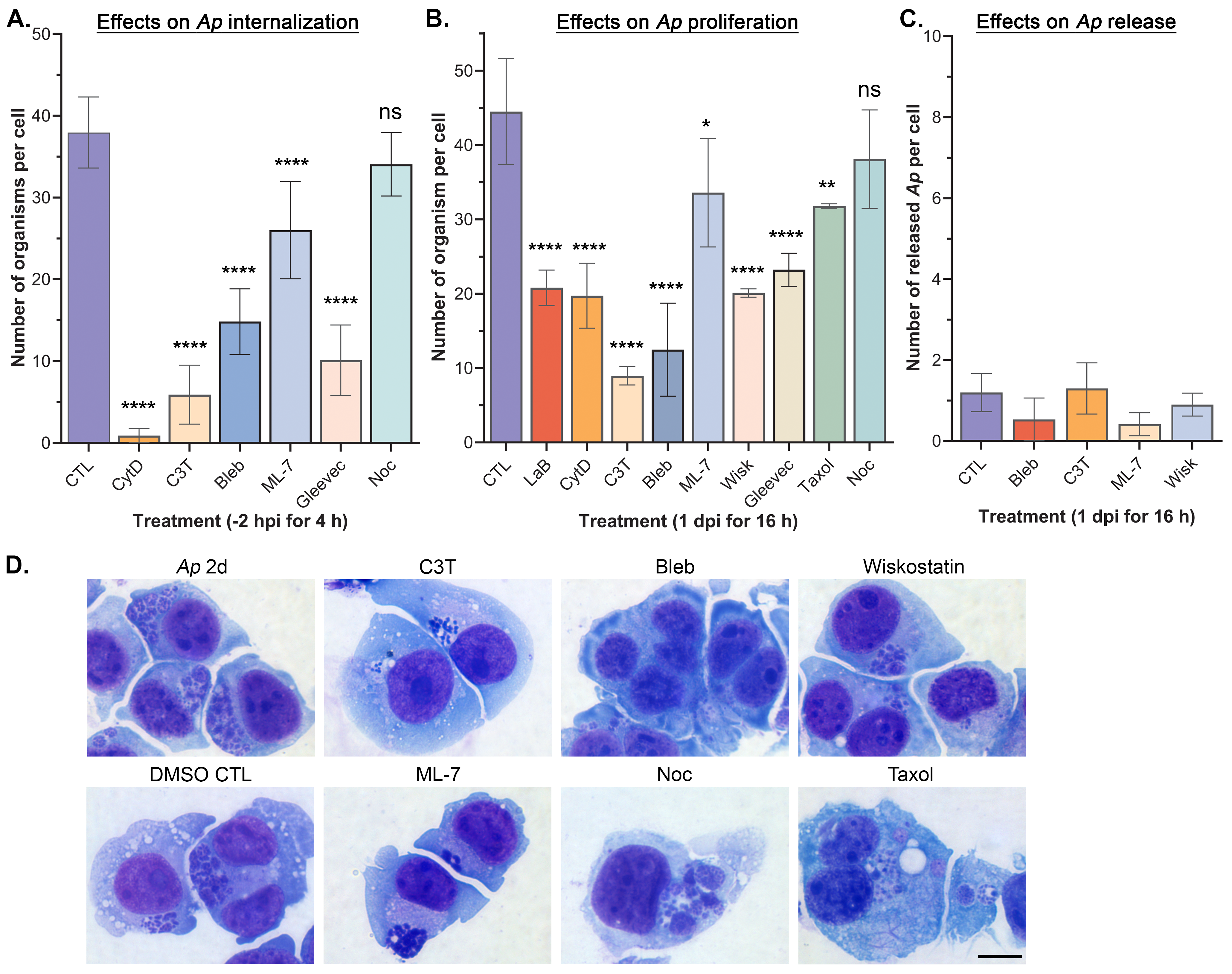

Supplement: S7 Fig — (A) To examine effects of cytoskeleton-disruption on bacterial internalization, naïve HL-60 cells were pretreated with inhibitors for 2 h and infected with host cell-free A. phagocytophilum (Ap) for 4 h in the presence of inhibitors. Cells were washed to remove inhibitors as well as unbound or uninternalized bacteria. (B–D) Alternatively, A. phagocytophilum-infected HL-60 were treated with inhibitors or DMSO solvent control (CTL) at 1 d pi for 16 h to determine effects of inhibitors on bacterial proliferation and release. Inhibitors and abbreviations: F-actin polymerization (cytochalasin D, CytD; latrunculin B, LaB); Abl-1 kinase (Gleevec), Rho GTPase (C3-transferase, C3T), Myosin II (blebbistatin, Bleb), MLCK (ML-7), N-WASP (Wiskostatin, Wisk), or microtubules (nocodazole, Noc; Taxol). Cells were cytospun onto slides for Diff-Quik staining, and infectivities were examined by counting numbers of intracellular Ap organisms in 100 cells in triplicates (A-B). Released Ap organisms either at extracellular spaces or attached to the cell surface (C) were quantified by counting approximately 80–100 cells. (A-C) Data were presented as the mean ± SD from three independent experiments. * P < 0.05; ** P < 0.01; **** P < 0.0001; ns, not significant; compared to the CTL group by one-way ANOVA. (D) Representative Diff-Quik staining images of panel B. Bar, 10 µm. (TIF) [file ppat.1014350.s008.tif]

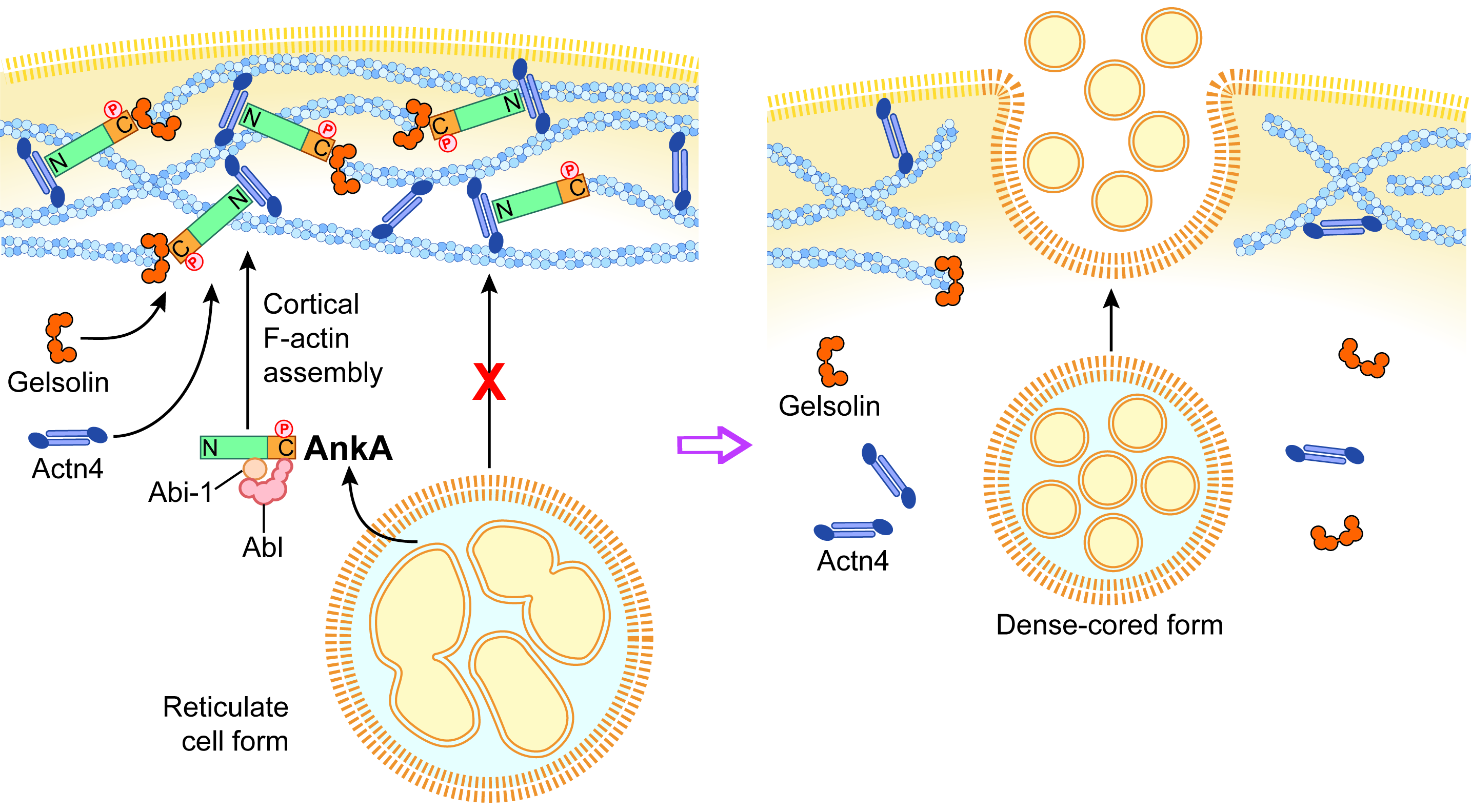

Supplement: S8 Fig — In proliferating (RC form)-stage of A. phagocytophilum-infected host cells, AnkA is secreted into host cells and interacts with Actn4, gelsolin, and actin at cell periphery, where the cortical F-actin network prevents the release of premature RC forms of A. phagocytophilum from host cells. The interaction of AnkA with host Abi-1 and Abl-1 tyrosine kinase, and subsequent AnkA phosphorylation are required for peripheral localization of AnkA and its interaction with F-actin. Mature DC forms of A. phagocytophilum, which turn down the expression of T4S apparatus and effector proteins including VirB9 and AnkA, could cause localized F-actin disassembly at cell periphery, thus inducing the release of mature A. phagocytophilum from infected cells. Illustrated by Tim Vojt. (TIF) [file ppat.1014350.s009.tif]
