# Supplementary material for: Obligatory intracellular bacterium Anaplasma phagocytophilum AnkA regulates actin dynamics and spatiotemporal bacterial release
Source: PLoS Pathog. 2026 Jun 24;22(6):e1014350. doi: 10.1371/journal.ppat.1014350 (PMC13293403; doi:10.1371/journal.ppat.1014350)
Supplement: S1 Table — (PDF) [file ppat.1014350.s010.pdf]

Table S1. Primer sequences for cloning of *A. phagocytophilum* AnkA protein and qPCR analysis

Gene Target	Primer sequence [1]	Purpose
**AnkA-N1**	F: 5'-GCTCGAATTC **ATG**TTGACAGAAGAAGAAAAG-3'<br>R: 5'-TAGACCGCGG TGGATCCGCAACATCTAC-3'	Cloning of *A. phagocytophilum* N-terminal fraction of AnkA (bp 1 ~ 2,610) into pET41a(+) expression vector at *EcoR*I and *Xhol* sites.
**AnkA-C1**	F: 5'-GCTCGAATTC TCATTGAAAACTCGTCCGTGG-3'<br>R: 5'-CATGCCGCGG CTACCTACCGCGACCTCC-3'	Cloning of *A. phagocytophilum* C-terminal fraction of AnkA (bp 2,611 ~ 3,699) into pET41a(+) expression vector at *EcoR*I and *Xhol* sites.
**AnkA-N2**	F: 5'-GCTCGAATTC **GCCACC *ATG*** **G**TGACAGAAGAAGAAAAG-3'<br>R: 5'-TAGACCGCGG TGGATCCGCAACATCTAC-3'	Cloning of *A. phagocytophilum* N-terminal fraction of AnkA (bp 1 ~ 2,610) into pEGFP-N1 mammalian expression vector at *EcoR*I and *Xhol* sites.
**AnkA-C2**	F: 5'-GCTCGAATTC **GCCACC *ATG*** **G**ATCCATCATTGAAAACTCG-3'<br>R: 5'-CATGCCGCGG CCTACCGCGACCTCCTTTTAC-3'	Cloning of *A. phagocytophilum* C-terminal fraction of AnkA (bp 2,611 ~ 3,699) into pEGFP-N1 mammalian expression vector at *EcoR*I and *Xhol* sites.
***Anaplasma* 16S rRNA**	F: 5'-GGTGAGTAATGCATAGGAATC-3'<br>R: 5'-GCTCATCTAATAGCGATAAATC-3'	qPCR and RT-qPCR analysis of *A. phagocytophilum* 16S rRNA gene [2]
**Human GAPDH**	F: 5'-AGCAATGCCTCCTGCACCACCAAC-3'<br>R: 5'-CCACATCACCCCTCTACCTC-3'	qPCR analysis of human *GAPDH* gene for HL-60 cells [2]
**Monkey GAPDH**	F: 5'-AGCAATGCCTCCTGCACCACCAAC-3'<br>R: 5'-CCGGAGGGGCCATCCACAGTCT-3'	RT-qPCR analysis of monkey *GAPDH* gene for RF/6A cells

1 F, forward primer; R, reverse complement primer; underlined, restriction enzyme sites; bold, Kozak sequences for mammalian cell expression. Full-length *ankA* gene was cloned by PCR amplification using AnkA-N forward and AnkA-C reverse primers.

2 Reference: Xiong Q, Lin M, Huang W, Rikihisa Y. Infection by *Anaplasma phagocytophilum* Requires Recruitment of Low-Density Lipoprotein Cholesterol by Flotillins. mBio. 2019;10(2): e02783-18. doi: 10.1128/mBio.02783-18.
