# Supplementary material for: Obligatory intracellular bacterium Anaplasma phagocytophilum AnkA regulates actin dynamics and spatiotemporal bacterial release
Source: PLoS Pathog. 2026 Jun 24;22(6):e1014350. doi: 10.1371/journal.ppat.1014350 (PMC13293403; doi:10.1371/journal.ppat.1014350)
Supplement: S1 Raw Images — (PDF) [file ppat.1014350.s012.pdf]

Fig. 3A

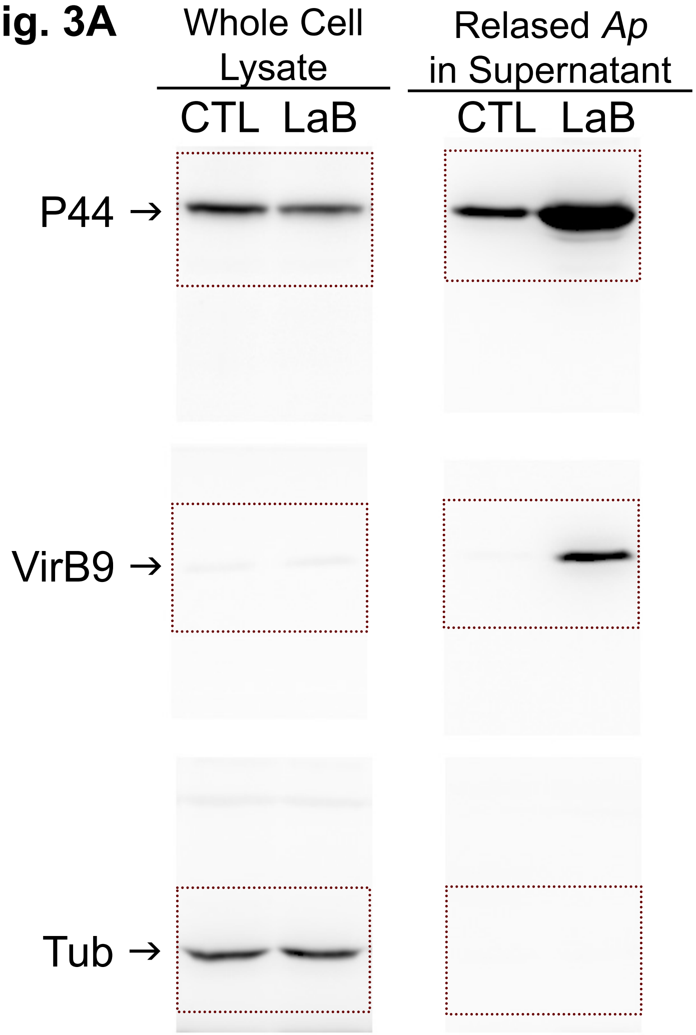

Whole Cell Lysate
Relased *Ap* in Supernatant
CTL
LaB
CTL
LaB
P44
VirB9
Tub

Fig. 4E

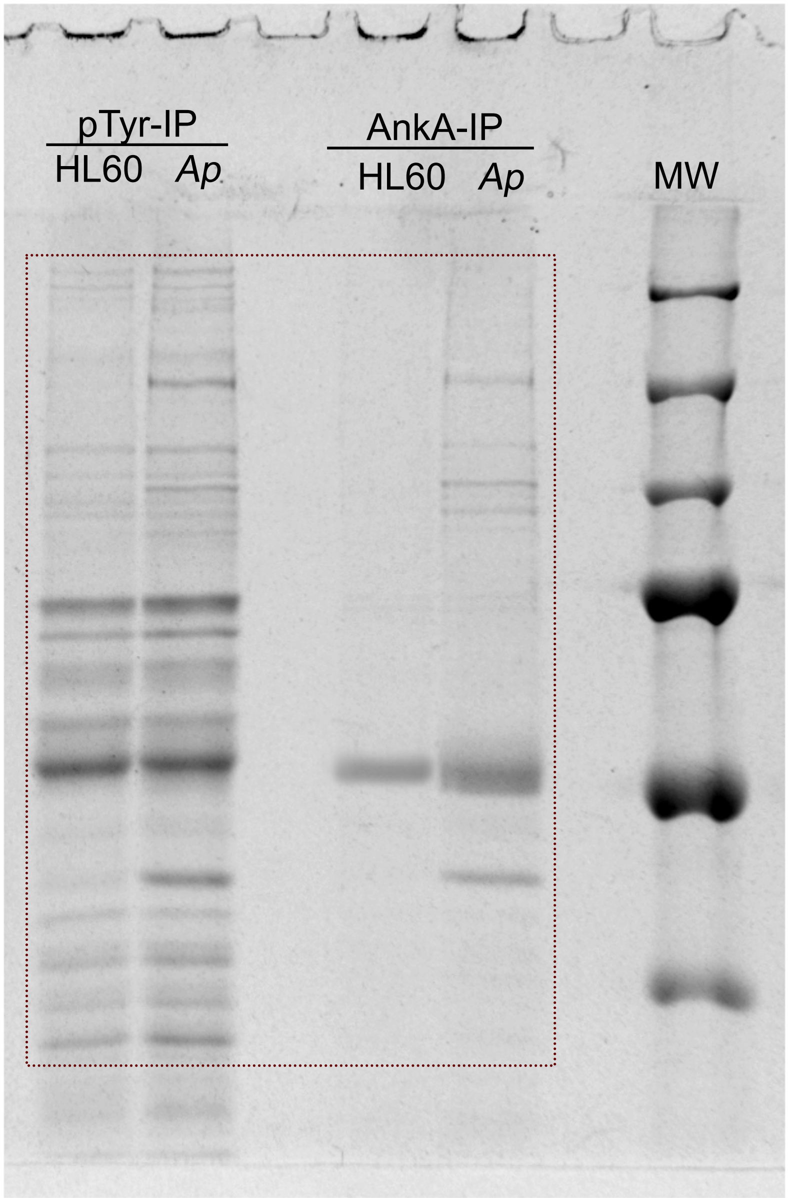

Fig. 4F

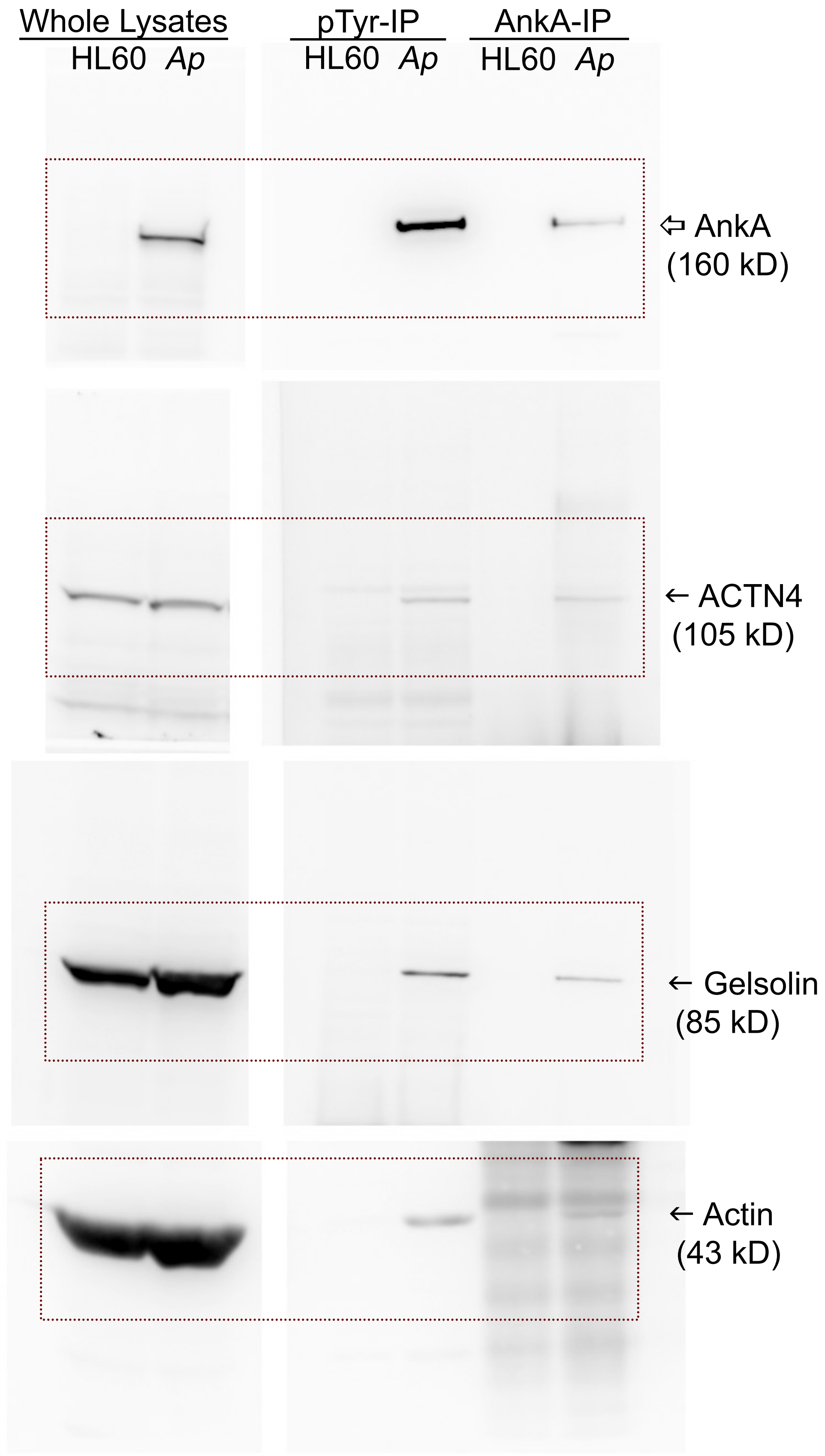

Whole Lysates
pTyr-IP
AnkA-IP
HL60 Ap
HL60 Ap
HL60 Ap
AnkA (160 kD)
ACTN4 (105 kD)
Gelsolin (85 kD)
Actin (43 kD)

Fig. 5A

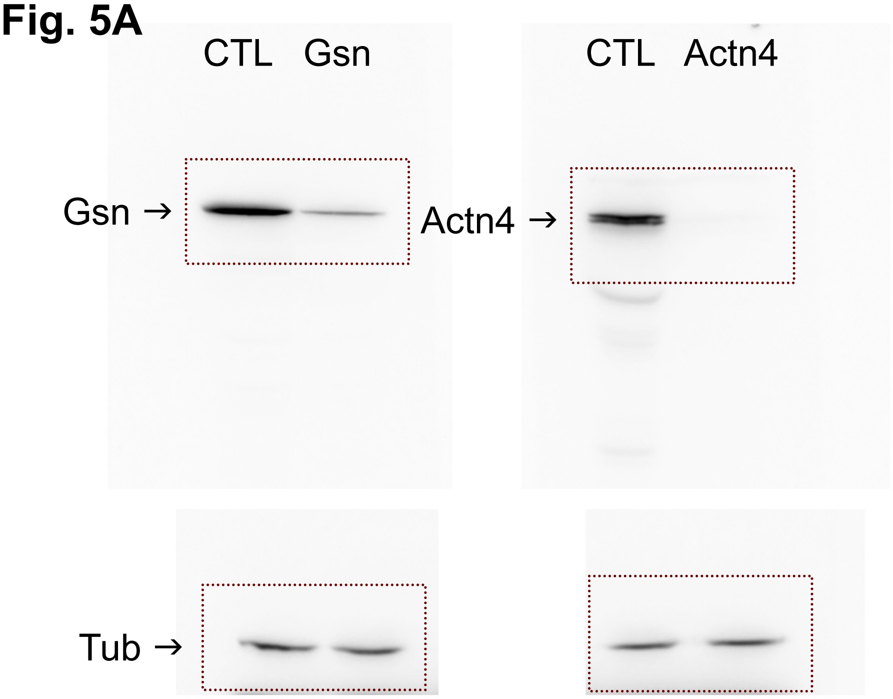

CTL Gsn
CTL Actn4
Gsn →
Actn4 →
Tub →

Fig. 5B

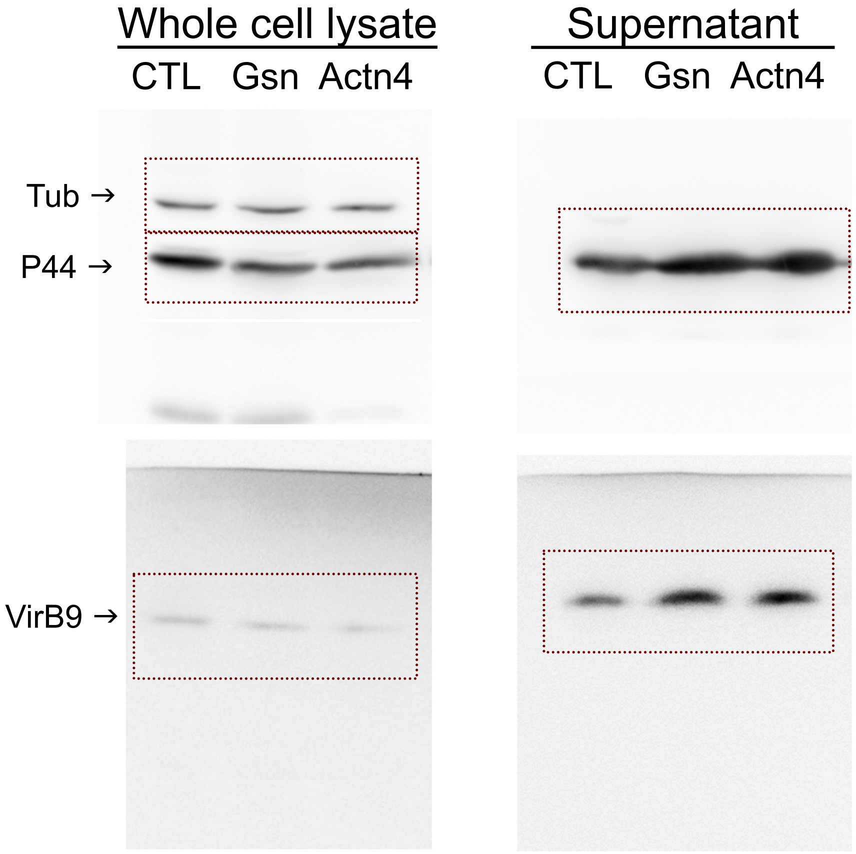

Whole cell lysate
Supernatant
CTL Gsn Actn4
CTL Gsn Actn4
Tub →
P44 →
VirB9 →

Fig. 6B

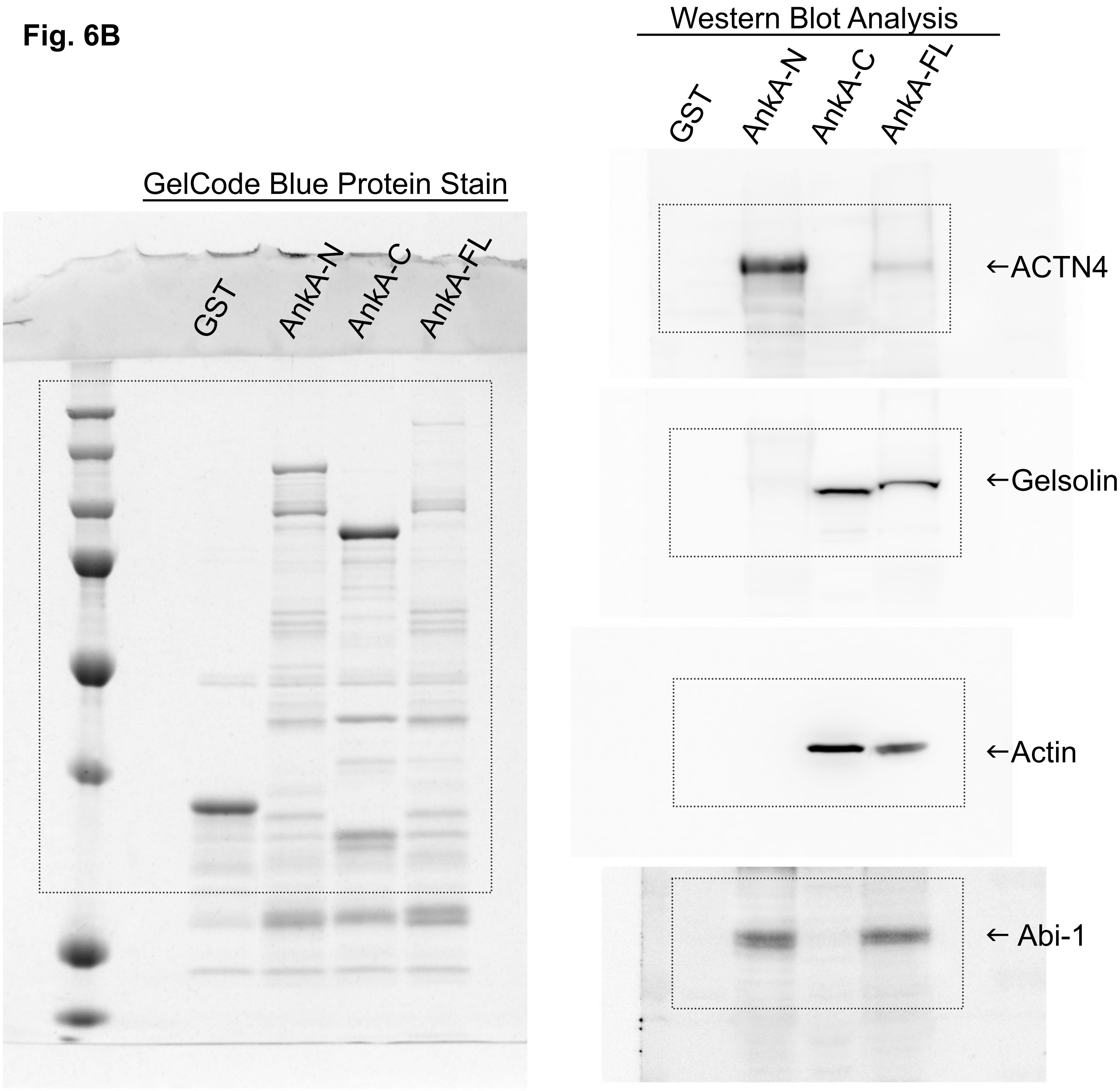
GelCode Blue Protein Stain
GST
AnkA-N
AnkA-C
AnkA-FL
Western Blot Analysis
GST
AnkA-N
AnkA-C
AnkA-FL
←ACTN4
←Gelsolin
←Actin
← Abi-1

Fig. 8A

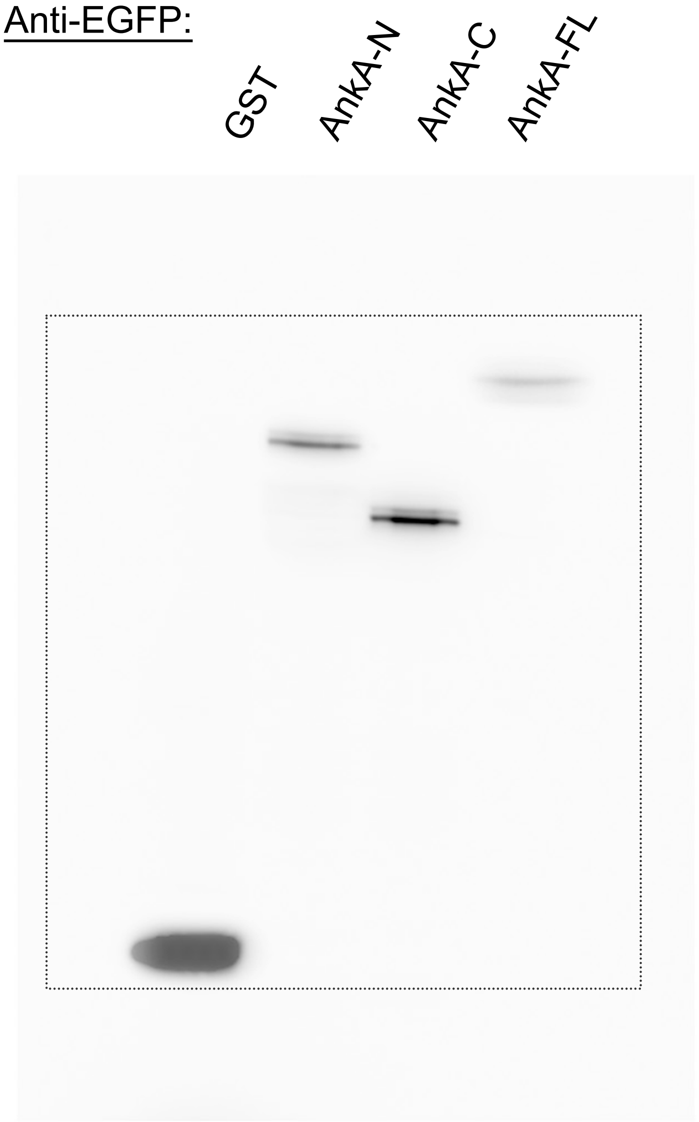

Anti-EGFP:
GST
AnkA-N
AnkA-C
AnkA-FL

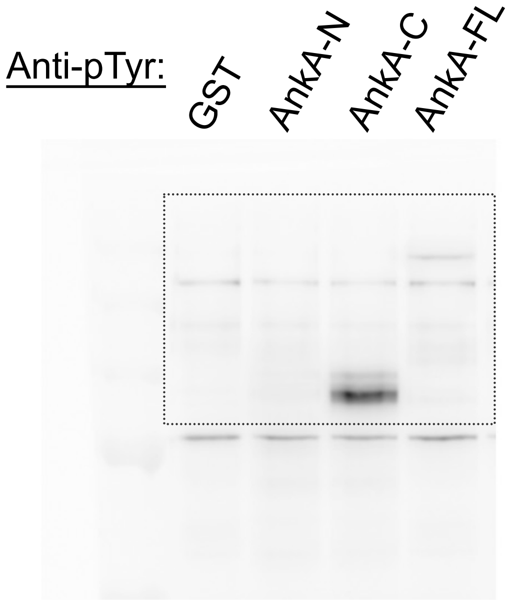

Anti-pTyr:
GST
AnkA-N
AnkA-C
AnkA-FL

Fig. 8B

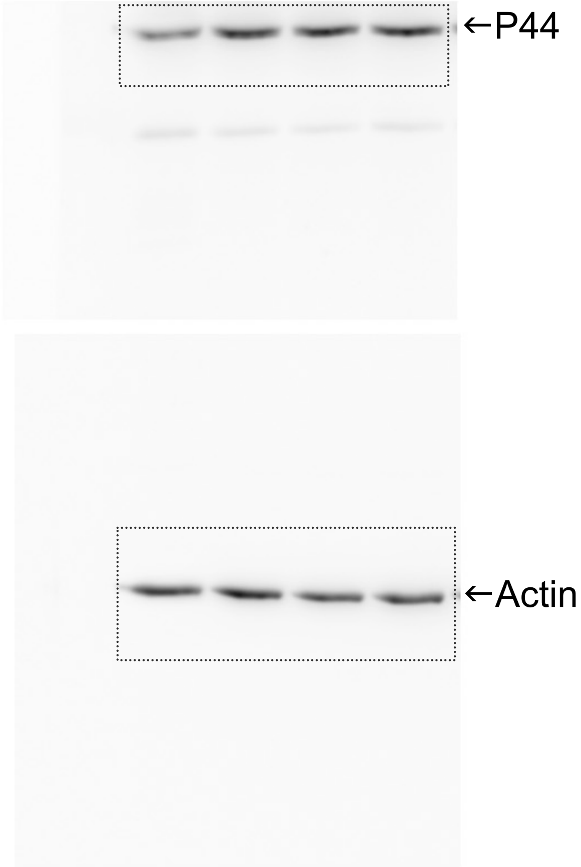

←P44
←Actin
